# Epigenetic repression of *CHCHD2* enhances survival from single cell dissociation through attenuated Rho A kinase activity

**DOI:** 10.1007/s00018-023-05060-8

**Published:** 2024-01-12

**Authors:** Jumee Kim, Eun-Ji Kwon, Yun-Jeong Kim, Dayeon Kim, Yoon-Ze Shin, Dayeon Gil, Jung-Hyun Kim, Hyoung Doo Shin, Lyoung Hyo Kim, Mi-Ok Lee, Young-Hyun Go, Hyuk-Jin Cha

**Affiliations:** 1 College of Pharmacy, Seoul National University, 1 Gwanak-ro Gwanak-gu, Seoul, 08826 Republic of Korea; 2 Research Institute of Pharmaceutical Science, Seoul National University, 1 Gwanak-ro Gwanak-gu, Seoul, 08826 Republic of Korea; 3 Korea National Stem Cell Bank, Osong, Republic of Korea; 4 Division of Intractable Disease Research, Department of Chronic Disease Convergence Research, Korea National Institute of Health, Osong Health Technology Administration Complex 202, Osong, Republic of Korea; 5 Department of Life Science, Sogang University, Seoul, Republic of Korea; 6 Research Institute for Basic Science, Sogang University, Seoul, Republic of Korea; 7 Research Institute for Life Science, GW Vitek, Inc., Seoul, Republic of Korea; 8 Stem Cell Convergence Research Center, Korea Research Institute of Bioscience and Biotechnology, Daejeon, South Korea

**Keywords:** *CHCHD2*, Survival trait, Culture adaptation, Mitochondria-dependent cell death, Pluripotent stem cells, ROCK, 20q11.21

## Abstract

During in vitro culture, human pluripotent stem cells (hPSCs) often acquire survival advantages characterized by decreased susceptibility to mitochondrial cell death, known as “culture adaptation.” This adaptation is associated with genetic and epigenetic abnormalities, including TP53 mutations, copy number variations, trisomy, and methylation changes. Understanding the molecular mechanisms underlying this acquired survival advantage is crucial for safe hPSC-based cell therapies. Through transcriptome and methylome analysis, we discovered that the epigenetic repression of CHCHD2, a mitochondrial protein, is a common occurrence during in vitro culture using enzymatic dissociation. We confirmed this finding through genetic perturbation and reconstitution experiments in normal human embryonic stem cells (hESCs). Loss of CHCHD2 expression conferred resistance to single cell dissociation-induced cell death, a common stress encountered during in vitro culture. Importantly, we found that the downregulation of CHCHD2 significantly attenuates the activity of Rho-associated protein kinase (ROCK), which is responsible for inducing single cell death in hESCs. This suggests that hESCs may survive routine enzyme-based cell dissociation by downregulating CHCHD2 and thereby attenuating ROCK activity. These findings provide insights into the mechanisms by which hPSCs acquire survival advantages and adapt to in vitro culture conditions.

**Supplementary Information:**

The online version contains supplementary material available at 10.1007/s00018-023-05060-8.

## Introduction

Contrasting somatic cells, human pluripotent stem cells (hPSCs) have distinctive cellular and molecular properties that preserve the genomic integrity [1–3]. In particular, hESCs have active DNA repair mechanisms and are considerably susceptible to genotoxic stimuli [4, 5]. These characteristics cause rapid cell death induction upon DNA damage through mitochondria-mediated apoptosis, preventing progeny from inheriting mutations [1]. Mitochondria induce rapid cell death in hESCs upon DNA damage through ‘high mitochondrial priming’ [6] due to high pro-apoptotic gene expression [7, 8] and the immediate active BAX translocation from the Golgi complex upon genotoxic insults [9]. In addition, when DNA damage occurs, cytoplasmic p53 stabilizes and moves into the mitochondria, increasing mitochondrial membrane permeability to induce swift apoptosis [7, 10]. Consistently, mutation accumulation of hPSCs is lower than somatic stem cells, when cultured in vitro [11].

Genetic abnormalities, such as recurrent copy number variations (CNVs) [12, 13] and chromosomal abnormalities [14] as well as abnormal mitosis [15], frequently arise when hPSCs are maintained in vitro, compromising hPSC-based cell therapy safety [16, 17]. Notwithstanding, the clinical consequences and underlying mechanisms of culture-induced (epi)genetic abnormalities in hPSCs remain uncertain [17]. Survival trait acquisition, known as culture adaptation [14], is a common phenotype during long-term culture [18–20] associated with not only *BCL2L1* induction [20] and recurrent CNV at 20q11.21 [18, 19, 21], but also dominant negative TP53 mutations [22], which impede the mitochondrial apoptosis. In addition, high mitochondrial priming of hESCs, through NOXA depletion, *BCL2L1* induction [8] or caspase inhibition [15], augments aneuploid populations, suggesting that survival trait acquisition during in vitro culture leads to further genetic alterations as previously proposed [23]. Accordingly, mutations that randomly occur in vitro culture favor culture-induced stress survival and the mutant clone becomes dominant to win the competition [23]. Such clonal dominance is attained through genetic variations with vigorous YAP activity [24] from a high TPX2 expression [25]. Furthermore, epigenetic aberrations including DNA methylation change, loss of parental imprinting, and variable X chromosome inactivation have also been reported in the prolonged culture of hPSCs [26]. Specifically, abnormal hypermethylation has been observed in tumor suppressor genes [27]and antioxidant genes [28]. However, only limited studies have been conducted to examine the biological consequence of these epigenetically repressed genes [29].

Coiled-coil-helix-coiled-coil-helix domain containing 2 (*CHCHD2*), coded by the *CHCHD2* gene (located on human chromosome 7p11.2), encodes a bi-organelle protein located in the mitochondria and nucleus [30]. The roles of this protein are multifaced, serving as a regulatory protein for mitochondrial energy metabolism and apoptosis, while also functioning as a transcription factor [31]. The significance of *CHCHD2* extends to various diseases. It has been implicated in cancers (i.e*.,* induction) [32, 33], Parkinson’s disease (i.e.*,* mutations) [34] and mitochondrial encephalomyopathy (i.e*.,* repression). However, the complete scope of *CHCHD2* functions remains to be fully determined [30]. Mitochondrial *CHCHD2*, initially identified as a mitochondrial chaperone protein binding partner, inhibits apoptosis through BCL-xL interaction in cancer cell models [35] and regulates mitochondrial morphology in *Drosophila* models [36]. Interestingly, *CHCHD2* expression in human embryonic stem cells (hESCs) primes neuroectodermal differentiation by sequestering SMAD4 in the mitochondria [37].

This study demonstrated that repeated hESCs enzymatic dissociation led to epigenetic repression of *CHCHD2*, closely associated with increased survival under diverse stresses encountered during in vitro culture. In addition, this effect was mediated through Rho A-ROCK signaling modulation. Consequently, hESCs lacking *CHCHD2* expression exhibited clonal dominance during long-term in vitro culture. These results suggest that loss of *CHCHD2* occurring through enzymatic dissociation is another cellular adaptation to favor cell survival under conditions of culture stress.

## Materials and method

### Cell culture

hESCs [WA09 (H9); WiCell Research Institute], BJ-iPSCs [38], and *CHCHD2* KO/reconstitution subclones were maintained in mTeSR1 (Stem Cell Technology, #85,851) or MACS-iPSC brew medium (Milltany Biotechnology, #130-104-368) on plates coated with Matrigel (Corning, #354,277) diluted at 1:80 in hESC basal medium (DMEM/F12 supplemented with 1% non-essential amino acids, 0.1% β-mercaptoethanol, and 0.1% gentamicin, Gibco) for feeder-free conditions. Cells were incubated in 37 °C, 5% CO_2_ conditions. The medium was replaced every day up to passaging, and the cells were enzymatically dissociated using a dispase solution (Gibco, #17,105,041) with 10 μM of Y-27632 (Biogems, #1,293,823). 1 ~ 2 × 10^5^ cells were seeding in 60Ø plate for transfer. 4 h mitomycin C-treated mouse embryonic fibroblast (MEF) was used as feeder cells for H9 feeder culture. Inverted microscope [Olympus, CKX-41 (Light source: 6 V/30W halogen lamp, Software: ProgRes Capture Pro)] was used to capture images for live cells.

### RNA extraction, quantitative real-time PCR, and genome-wide gene expression profiling

Total RNA was extracted using Easy-blue reagent (Intron, #17,061) in accordance with the manufacturer’s instructions. PrimeScript™ RT reagent kit (Takara, #RR014A) was used to generate total RNA to cDNA following the manufacturer’s protocol. Quantitative real-time PCR (qPCR) was performed using TB green premix Taq (Takara, #RR820A) on a LightCycler 480 Instrument II (Roche) according to the manufacturer’s instructions. Primer information is shown in Table 1.

**Table 1:** Primer sequences for real-time PCR analysis

Gene	Forward sequence (5ʹ to 3ʹ)	Reverse sequence (5ʹ to 3ʹ)
*CHCHD2*	CAGCAGCCTTGCCTCTATG	GTTTGCAAGTCGGCACTGT
*BCL2L1*	GATCCCCATGGCAGCAGTAAAGCAAG	CCCCATCCCGGAAGAGTTCATTCACT
*POU5F1*	GTGGAGGAAGCTGACAACAA	ATTCTCCAGGTTGCCTCTCA
*SOX2*	TTCACATGTCCCAGCACTACCAGA	TCACATGTGTGAGAGGGGCAGTGTGC
*T*	CAGTGGCAGTCTCAGGTTAAGAAGGA	CGCTACTGCAGGTGTGAGCAA
*PAX6*	TGTCCAACGGATGTGTGAGT	TTTCCCAAGCAAAGATGGAC
*NES*	TCCAGAAACTCAAGCACCA	AAATTCTCCAGGTTCCATGC
*OTX2*	CAGCAACAACAGCAGAATGGAGGT	TGGCCACTTGTTCCACTCTCTGAA
*SOX17*	AGCAGAATCCAGACCTGCAC	TTGTAGTTGGGGTGGTCCTG
*ID1*	CTGCACACCTACTAGTCACCAGAG	CAGAAATCTGAGAAGCACCAAACGTG
*SLC35F2*	TGTTGGACTCTTTCTGTTTGGC	GGTCTCCTGGAGGTTCTCCT
*18S*	GTAACCCGTTGAACCCCATT	CCATCCAATCGGTAGTAGCG

For library construction, we used the TruSeq Stranded mRNA Library Prep Kit (Illumina, San Diego, CA). Briefly, the strand-specific protocol included the following steps: (1) strand cDNA synthesis, (2) strand synthesis using dUTPs instead of dTTPs, (3) end repair, A-tailing, and adaptor ligation, and (4) PCR amplification. Each library was then diluted to 8 pM for 76 cycles of paired-read sequencing (2 × 75 bp) on an Illumina NextSeq 500 following the manufacturer’s recommended protocol. Read quality was assessed using FastQC (v) and poor-quality bases (Phred score < 20) were eliminated using TrimGalore (v0.6.6). Trimmed reads were aligned to the human reference genome (GRCh38) using the STAR aligner (v2.7.9a) with default parameters. Gene-level expression values such as transcripts per million (TPM) and read counts were calculated using RSEM (v1.3.3.) with human gene annotation (GRCh38.84). FASTQ format files, gene-level count data, and TPM of all samples are available in the Gene Expression Omnibus. Differential gene expression analysis for P1 ~ P4 data was performed using the ‘DESeq2’ package (v3.15) in R (v4.2.1). Differential gene expression analysis for *CHCHD2* KO data was performed using the ‘EdgeR’ package (v3.15) in R (v4.2.1). Transcripts were considered significant if their fold change was above or below 1 (in log2 scale) and the p value corrected by FDR was below 0.05.

### Chromatin immunoprecipitation (ChIP)

Cells were cross-linked with 1.21% formaldehyde for 10 min, and then quenched with Glycine for 5 min at room temperature. After fixation, chromatin samples were sonicated for 10 s 25 times to generate 300–500 bp fragments. Chromatin was immunoprecipitated with antibodies against rabbit IgG (Invitrogen, #31,235), H3K9me3 (CST, #13,969), H3K27me3 (CST, #9733), and H3K27Ac (Abcam, #ab4729). The eluted DNA was purified with phenol–chloroform. ChIP-qPCR was performed for quantification using *CHCHD2* primers (F: 5ʹ-CAG GCC TGA AGT TCA TTG GAA-3ʹ, R: 5ʹ-ACT TCC GGG TTT TAA AGA TCC T-3ʹ), following the qRT-PCR protocols above.

### Teratoma forming assay

For the teratoma forming assay, H9-hESCs with CHCHD2 wild type and knockout were cultured for 4 days. Cells were dissociated by accutase and counted as 1 × 10^6^ cells for injection. The cells were injected into the right testis of 4-weeks-old BALB/c-nude mice along with Y-27632 in mTeSR1 medium. The left testis was used as the control. After 4 weeks, the first teratoma formation was observed, and after an additional 2 weeks, the mice were killed and the teratoma was extracted. A paraffin block was made using extracted teratoma, and H&E staining was performed.

### Promoter methylation analysis using endonuclease digestion

For DNA methylation analysis in CHCHD2 promoter regions, genomic DNAs were extracted using genomic DNA purification kit (Promega, #A1120) according to the manufacturer’s instructions. After the extraction, 1 μg of genomic DNA was digested by methylcytosine-sensitive endonuclease McrBC (Takara, #1234A) for 4 h at 37 °C and inactivated at 65 °C for 20 min. Genomic DNAs without McrBC with the same reaction and amount were used as a control. Samples after digestion were diluted to a final volume of 400 μl, and 9 μl for each qRT-PCR was used. Primers were designed to be located in CpG island of the *CHCHD2* promoter region. Sequence information of forward and reverse primers was as follows: F: 5ʹ-GTGAGTCACTCTTAAGGTTGGA-3ʹ, R: 5ʹ-AAGAGCTAAGCGACTTCTGAG-3ʹ.

### Temporal trend classification of gene expression

Gene expression was normalized using *Z*-score or mean-centering normalization. *Z*-score was applied after collapsing all replicates to their average at each time point. Next, we calculated gene distances using Euclidean distance and used complete clustering methods.

### Re-analysis of published transcriptomics data

The current publication utilized processed data from the following publications: Garitaonandia et al. (GSE34982). Data was downloaded directly from GEO repository. Transcript intensities after processing were log2 transformed and normalized using “Loess” (from limma package in R). After that, the R limma program was used to estimate the difference genes between groups. Transcripts were considered differentially expressed if their fold change was above or below 1 (in log2 scale) and the p value corrected by FDR was below 0.01.

### Determination of copy number variations

Whole-genome genotyping was performed using the Illumina HumanOmni1-Quad Beadchip (Illumina) containing 1,140,419 genetic markers across the human genome. Samples were processed according to the specifications of the Illumina Infinium HD super assay. Briefly, each sample was whole genome amplified, fragmented, precipitated, and re-suspended in an appropriate hybridization buffer. Denatured samples were hybridized on a prepared BeadChip for a minimum of 16 h at 48 °C. Following hybridization, the bead chips were processed for the single-base extension reaction, stained, and imaged on an Illumina iScan system. Normalized bead intensity data for each sample were loaded into the GenomeStudio software package (Illumina). Ratios of signal intensity were calculated using the Log R Ratio (LRR: logged ratio of observed probe intensity to expected intensity; any deviations from zero in this metric are evidence for copy number change) and allelic intensity was determined by the B allele frequency for all samples. Values were exported using Illumina GenomeStudio. Analysis for structural variants was performed using the sliding window approach (window size 10).

### Re-analysis of published methylation profile data

The current publication utilized processed data from the following publications: Garitaonandia et al., (GSE34982). Methylation profiles were downloaded as β values directly from the GEO repository. By range scaling to control samples of unmethylated, fully methylated, and half-methylated DNA, methylation levels for each probe were produced [39]. Methylation differences were calculated by subtracting the *ß*-values. For instance, if the difference in methylation was larger than 0.2 and the change in expression was greater than 2, an included gene of these category was deemed to be hypermethylated and silenced.

### Immunoblotting and immunofluorescent assay

Immunoblotting and immunofluorescent assay were performed as described previously [40]. Antibody for *CHCHD2* (#19,424-1-AP) was purchased from Proteintech. Antibodies for cleaved caspase-3 (C.Casp-3, #9664), OCT-4A (#2840), pPTEN (#9551), and pMYPT1 (#5613) were purchased from Cell Signaling Technology. Antibodies for α-tubulin (#sc-5286) and β-actin (#sc-47778) were purchased from Santa Cruz Biotechnology. Antibody for BCL-xL (#ab32370) and phosphor-BAX (#ab111391) were purchased from Abcam. Antibodies for Flag (#F1804) and active BAX (#MABC1176M) were purchased from Sigma-Aldrich. Quantification of blots was performed by Fusion software (Vilber Lourmat) in accordance with the manufacturer’s protocol. For immunofluorescent assay, secondary antibodies for mouse primary antibody conjugated to Alexa Fluor 488 (A11029) and Alexa Fluor 594 (A11032) fluorophores as well as for rabbit primary antibody conjugated to Alexa Fluor 488 (A11034) and Alexa Fluor 594 (A11037) were purchased from Invitrogen. Nucleus staining reagent 4’,6-diamidini-2-phenylindole (DAPI, #D1306) was purchased from Thermo Fisher Scientific. Fluorescence microscopy [Olympus, BX53 (Light source: 103W mercury lamp/12V 100W halogen lamp, Software: CellSense)] was used for imaging samples.

### Immunoprecipitation

For immunoprecipitation, 1 mg of total proteins was incubated with 2 μg of *CHCHD2*, BCL-xL, or Flag antibody at 4 °C for 16 h, followed by the addition of Protein-A or G agarose beads (Santa cruz, #sc-2001 and #sc-2002) and incubated at 4 °C for an additional 4 h. The precipitates were washed with tissue lysis buffer (TLB) for 15 min each more than three times, followed by immunoblotting performed in accordance with the protocol reported previously [40] using second HRP antibody (Jackson Laboratory, #111-035-003 and #115-035-003) or Variblot (Abcam, #ab131366).

### Alkaline phosphatase (AP) assay

Alkaline phosphatase (AP) staining assay was performed as per the manufacturer’s protocol (Sigma-Aldrich, #SCR004), and bright field images were captured by digital single-lens reflex camera (Nikon, D80).

### Mitochondrial isolation

Mitochondrial isolations from human embryonic stem cells were performed by mitochondria isolation kit for cultured cells (ThermoFisher, #89,874) according to the manufacturer’s instructions. Mitochondria lysis for immunoblot was preserved by RIPA buffer or 2% CHAPS in TBS as described elsewhere.

### Flow cytometry

For antibody staining flow cytometry, cells were washed twice with PBS and ixed with fix-permeabilization solution (BD Bioscience, #554,722). Cells were washed with permeabilization-wash solution twice (BD Bioscience, #554,723) and stained with primary antibodies in 3% BSA solution for 1 h, followed by 1 h of incubation with fluorescent-conjugated secondary antibodies.

### Clonogenic assay

For the clonogenic assay, human embryonic stem cells were detached as single cell by accutase in 37 °C CO_2_ incubator for 3 min. Single cell-dissociated cells were seeded 5 × 10^4^ cells for each wall into Matrigel-coated six-wall plate and cultured 5 ~ 7 days. After culture, 4% paraformaldehyde (PFA) solution was used for 10 min for fixing, and 0.1% of crystal violet solution was used for 1 h for staining the cells. After the crystal violet staining, the dye was destained using distilled water (DIW) twice and dried overnight to remove residual humidity. Colony images were captured by an optical camera (Nikon #NKR-D80(B)) and analyzed by Image J software.

### Cell death analysis

Cell death was analyzed by flow cytometry as described previously [25]. For Annexin V/7-AAD staining, cells at 24 h after treatment of each flavonoid were washed twice with PBS and stained with FITC-conjugated Annexin V antibody (BD Bioscience, # 556,419) and 7-AAD (BD Bioscience, #559,925) for an additional 45–60 min at room temperature in the dark. Cells stained with Annexin V/7-AAD were analyzed by FACS Calibur or FACS Lyric (BD Bioscience). For the bright field images captured, light channel of optical microscope (Olympus, CKX-41) or JuLI-stage (NanoEntek, Korea) was used in accordance with the manufacture’s protocol. JuLI-STAT was used for analysis of the data from JuLI-stage (NanoEntek, Korea). The activity of caspase-3 was analyzed by colorimetric active caspase-3 assay kit (Sigma-Aldrich, #CASP3C) in accordance with the manufacturer's protocol.

### Construction of *CHCHD2* sgRNA and knockout hESCs

To construct the *CHCHD2* knockout (KO) hESCs, two sgRNAs targeting for intron 2 and 3 to eliminate Exon 3 of *CHCHD2* were predicted by RGEN Tool (http://www.rgenome.net/cas-designer/). Two sgRNAs were cloned into pX330A and pRGEN-U6-GFP vector and transfected into H9-hESCs by electroporation with the CRISPR/Cas9 vector. Cells were double selected by GFP and puromycin and single cell cloning was performed for equal genetic background.

### Construction of doxycycline-inducible *CHCHD2* vector

To construct the doxycycline-inducible *CHCHD2* and rtTA piggybac plasmid, Flag-tagged (in C-terminal) human *CHCHD2* in pCL-Neo backbone vector, kindly provided by Dr. Lawrence I. Grossman, was cloned into piggybac plasmid pB-TET. Two piggybac plasmids were transfected into normal or long-term cultured H9 and CHA3 hESCs using electroporation (NEPA) and selected using a 200 μg/mL of G418 (Sigma-Aldrich, #A1720) 3 days later. After the G418 selection, Flag-tagged *CHCHD2* expressing a minimal dosage of doxycycline (Sigma-Aldrich, #D9891) was selected using colony picking, and single cell cloning was performed.

### Statistical analysis

The graphical and quantification data were presented as mean ± S.D. Statistical significance among the three groups and between groups was determined using one-way or two-way analysis of variance (ANOVA) following Tukey post-test and Student’s *t* test, respectively. Statistical analysis was performed with GraphPad Prism 8 software (https://www.graphpad.com/scientific-software/prism/). Significance was assumed for *p* < 0.05 (*), *p* < 0.01 (**), *p* < 0.001 (***), *p* < 0.0001 (****).

## Results

### Identifying *CHCHD2* as a potential hESCs repetitive culture biomarker

**Fig. 1:**
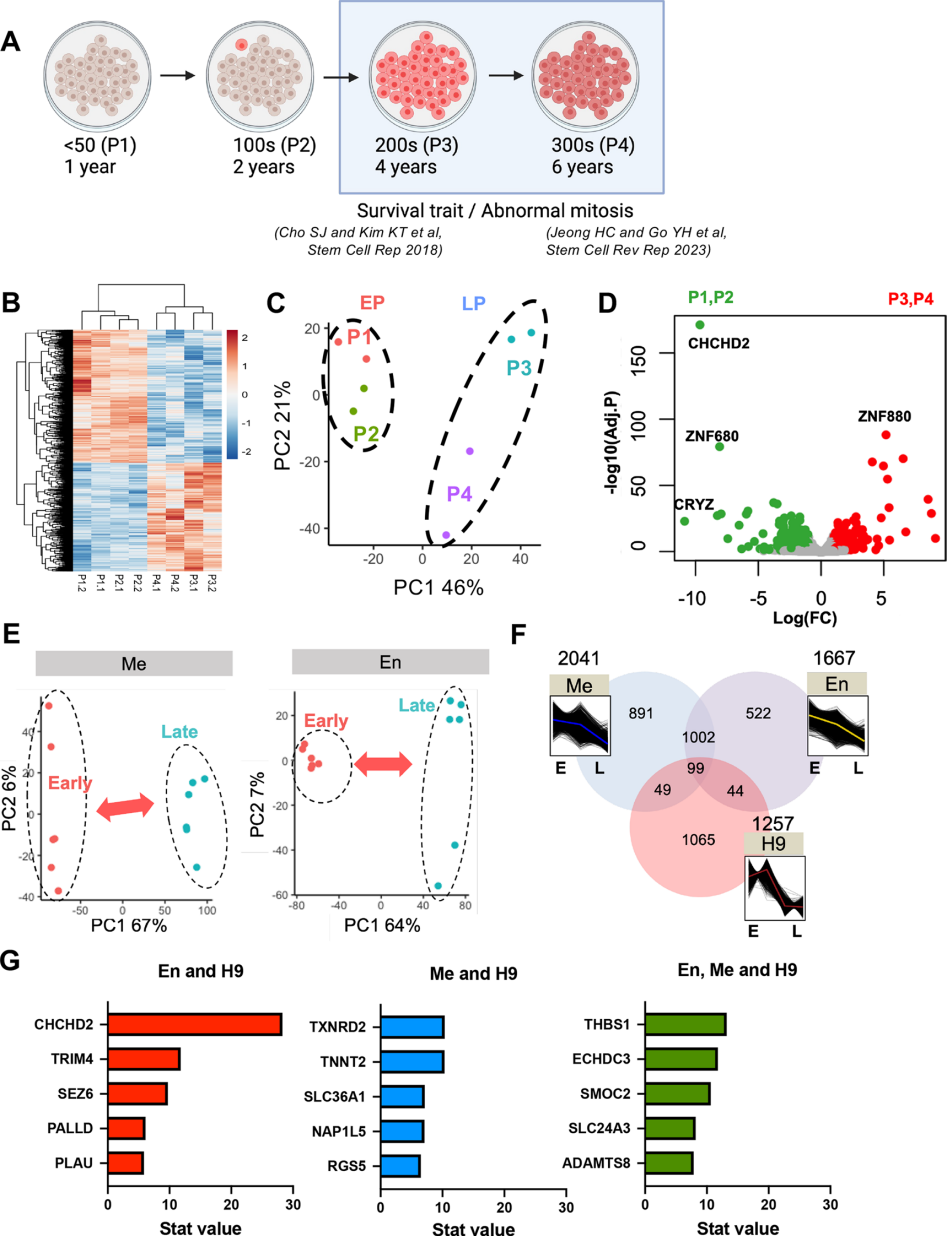

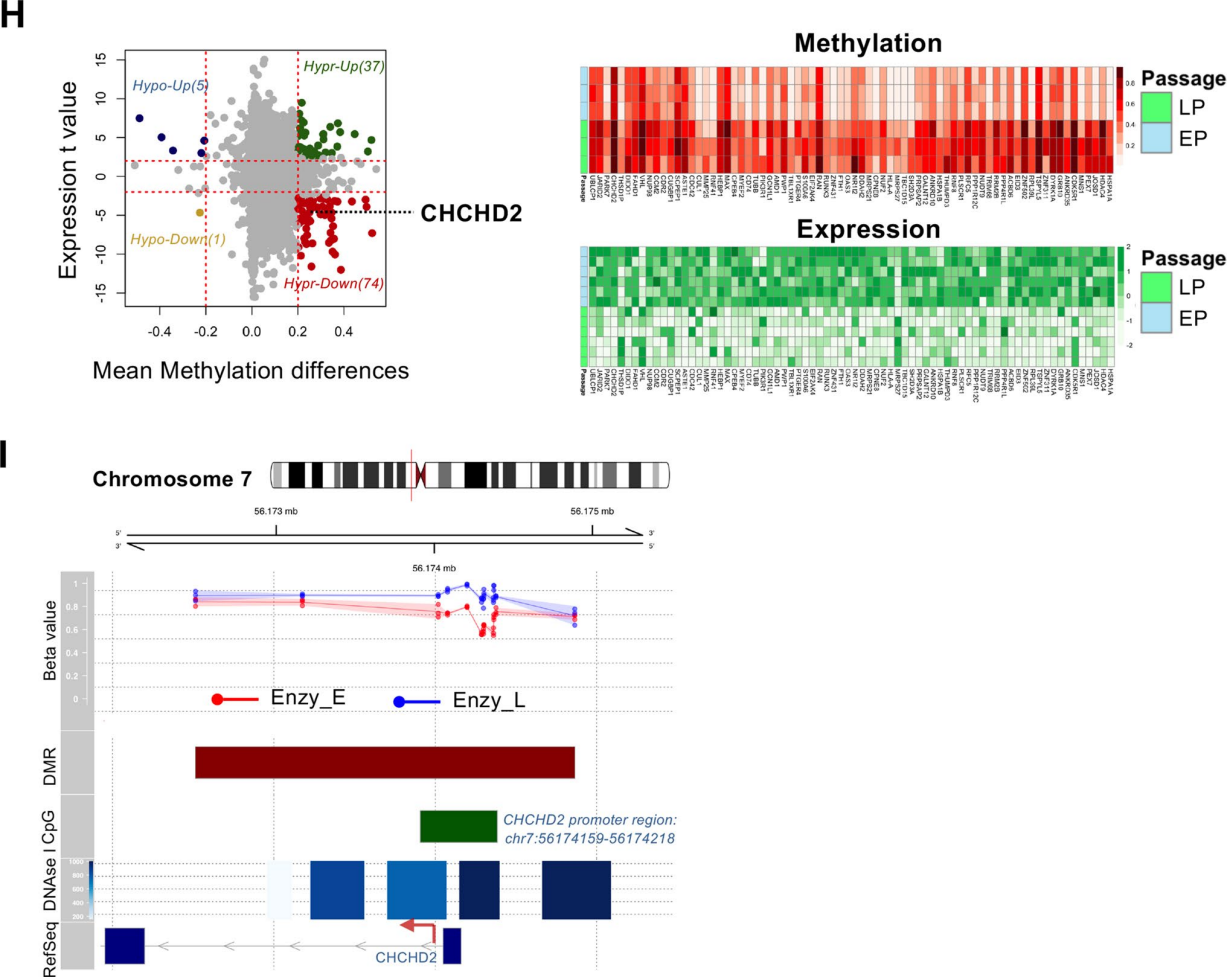
Identify *CHCHD2* as a potential gene marker of LP-hESCs. **A** Schematic images of long-term cultured hESCs model (P1: 40 s, P2: 100 s, P3: 200 s, P4: over 300 passages). **B** Heatmap of RNA sequencing for passage-dependent hESCs (*EP* early passage, *LP* late passage). **C** PCA analysis of RNA sequencing for passage-dependent hESCs. **D** Volcano plot of DEGs for passage-dependent hESCs. **E** PCA analysis of passage-dependent hESCs with enzymatic or mechanically cultured hESCs was displayed. **F** Diagram of downregulated intersection genes between passage and culture method. **G** List of downregulated genes for early passage and different culture methods (*Me* mechanical, *En* enzymatic) in hESCs. **H** Scatter plots of RNA expression and DNA methylation differences in high passage compared to low passage samples in data from GSE34982 (Hypo: low methylation, Hypr: high methylation, Up: high expression, Down: low expression) (left). The RNA expression and DNA methylation data of the probes in the Hyper-Down dataset showed opposite pattern, as shown in the heatmaps. (right). **I** Differentially methylated region (DMR) analysis at CHCHD2 promoter region of different passages with cultured enzymatically in data from GSE34982. Beta value, DMR, CpG islands, DNase clusters, and reference gene are shown below each plot

To identify potential long-term cultured [or late passage (LP)] hESC biomarkers, we capitalized on four passage-dependent H9 hESC variants (P1: < 50, P2: 100 s, P3: 200 s, and P4: 300 s passages), in which survival trait (or survival advantage) [20] and abnormal mitosis [15] were apparent after 200 passages (P3 and P4) (Fig. 1A). In addition, we performed RNA sequencing on these four hESC sublines to explore hESCs’ long-term culture effect on its gene expression. We observed a clear difference in overall gene expression (Fig. 1B) and distinct clustering (Fig. 1C) between early (EP cluster: P1 and P2) and late passages (LP cluster: P3 and P4). We indicated the differentially expressed genes (DEGs) via volcano plot, revealing the mostly distinct *CHCHD2* repression from upregulated and downregulated genes in LP-hESCs (Fig. 1D). To search for the ‘potential biomarker’ to reflect the culture adaptation, we utilized transcriptome datasets from one large-scale study (GSE34982) previously reported [41] to monitor potential effects of diverse culture conditions [passaging methods (mechanical vs. enzymatic) and culture matrices (extracellular matrix (ECM) vs. mouse feeder) for different passaging numbers (early vs. late)] (Fig. S1A). We further compared significantly altered gene expression under repetitive culture conditions [mechanical (Me) or enzymatic (En) dissociation], obtained from the dataset of GSE34982 (Fig. 1E) with transcriptome data from the in-house model (H9 hESCs) for early (E) and late (L) passage (Fig. 1F). Compared to altered LP-hESCs genes with transcriptome data from different culture conditions (Fig. 1F), CHCHD2 loss, distinctly observed in LP-hESCs (Fig. 1D), was a common repetitive enzymatic dissociation event (Fig. 1G).

The methylome data, previously reported to show significant epigenetic aberrations in long-term culture conditions [41], were further analyzed in detail. As described earlier [41], there were distinct alterations in methylation patterns at CpG sites were observed in hESCs that had been cultured for an extended period (Fig. S1B). Where a larger number of differentially methylated CpG (DMC) sites were observed in mechanically passaged hESCs over time in culture (Fig. S1B), a different methylation profile was observed between mechanical and enzymatic passaging methods (Fig. S1C). Notably, CHCHD2, hypermethylated in enzymatic passaging (Fig. S1D), was identified as one of the hypermethylated genes that exhibited strong repression, as shown in Fig. 1H. In more detail, differentially methylated region (DMR) analysis clearly revealed that hypermethylation occurred at the CHCHD2 promoter region in long-term enzymatic passaging (Fig. 1I).

### *CHCHD2* depletion during prolonged hESC culture

**Fig. 2:**
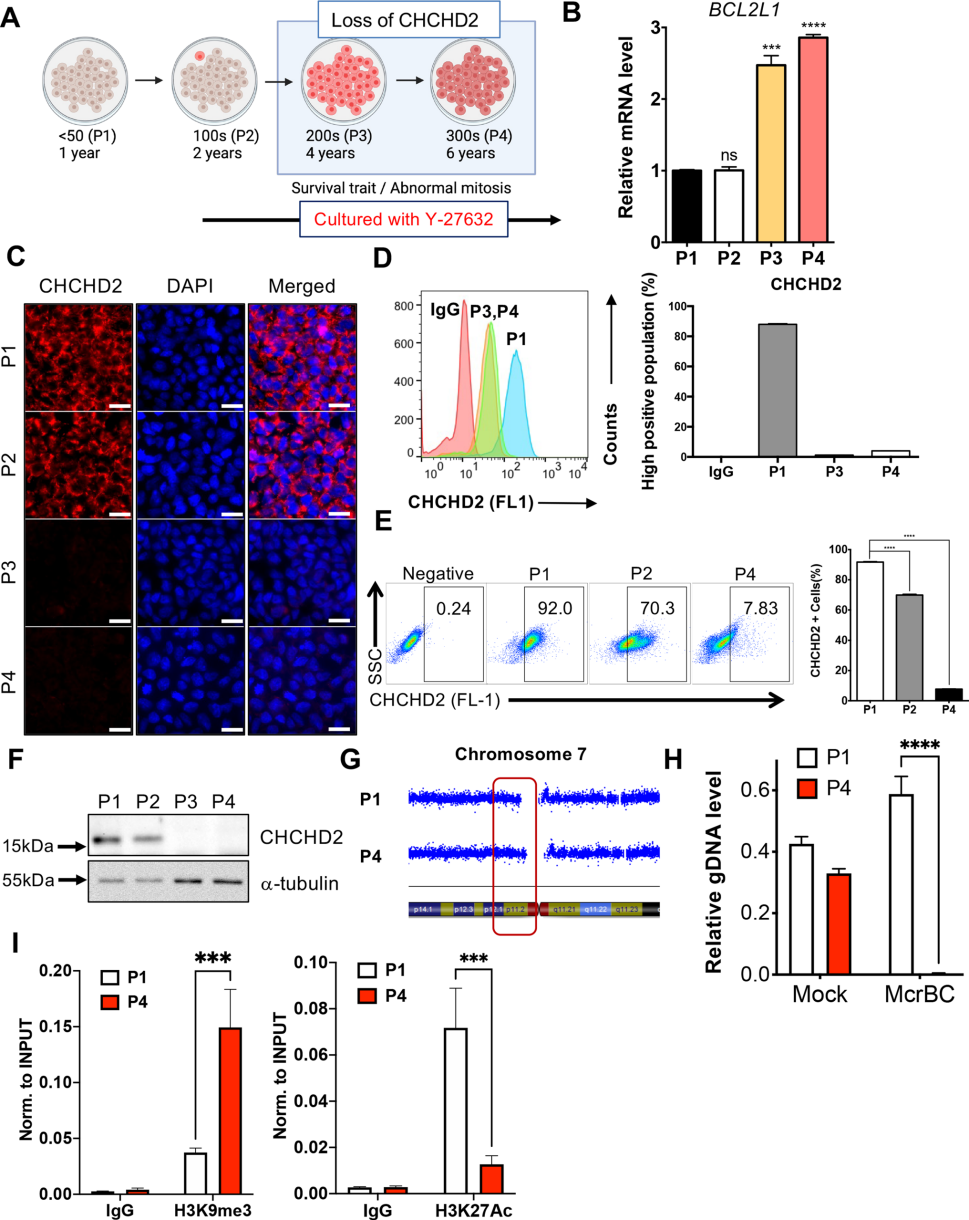
Depletion of *CHCHD2* in long-term cultured hESCs. **A** Schematic images of the LP-hESCs model cultured with ROCK inhibitor (Y-27632). **B** mRNA expression of *BCL2L1* in passage-dependent cultured hESCs (*n* = 2). **C** Immunofluorescent assay for CHCHD2 in passage-dependent cultured hESCs (scale bar = 25 μm). **D** Flow cytometry (left) and quantification graph (right) for CHCHD2 with fluorescent conjugated staining in low and high passage hESCs (P1: 40 s, P3: 200 s, P4: over 300 passages), and IgG for negative control of CHCHD2 primary antibody (*n* = 2). **E** Flow cytometry (left) and quantification graph (right) for CHCHD2 with fluorescent conjugated staining in passage-dependent cultured hESCs (P1: 40 s, P2: 100 s, P4: over 300 passages), and IgG for negative control of CHCHD2 primary antibody (*n* = 2). **F** Immunoblot assay for CHCHD2 protein expression in passage-dependent cultured hESCs, and α-tubulin for equal protein loading control. **G** Log R ratio (LRR) plot (i.e., a normalized measure of the total signal intensity for two alleles of the SNP) of P1 and P4 hESCs for chromosome 7 indicating the relative abundance of the genomic DNA around the SNP, which is expected to correlate with the copy number. **H** qRT-PCR-based methylation level analysis in the *CHCHD2* promoter region using the methylation-sensitive restriction enzyme McrBC. Samples are shown as a relative value to the *CHCHD2* exon (*n* = 3). **I** Chip-qPCR analysis of methylation (left) and acetylation (right) of two passage-dependent (early: P1, late: P4) hESCs in the *CHCHD2* promoter region (*n* = 2)

Notably, enzymatic dissociation has been used as hESCs culture methodology since the Rho-associated protein kinase (ROCK) chemical inhibitor (Y-27632) was identified to block single cell-induced cell death [42]. Before this report, P1 hESCs were maily maintained by mechanical dissociation, so that they were much less exposed to enzymatic dissociation with Y-27632 (Fig. 2A). As predicted in silico in Fig. 1, *CHCHD2* expression was drastically repressed in P3 and P4 hESCs where *BCL2L1,* a known marker for ‘culture adaptation’ or ‘survival advantage’ [20], was highly upregulated (Fig. 2B). Drastic *CHCHD2* protein repression in P3 and P4 hESCs was made observable through immunofluorescence (Fig. 2C) and flow cytometry (Fig. 2D). In P2 hESC, representing a part of the total 100s passages was carried out, enzymatic dissociation revealed a significant decrease in *CHCHD2* expression compared to P1 hESCs (Fig. 2E and F). The normal copy of 7p11.2, where *CHCHD2* is located, failed to account for the distinct repression of *CHCHD2* (Fig. 2G).

As similar as strong epigenetic repression of CHCHD2 (Fig. 1H) and their promoter region (Fig. 1I) by prolonged enzymatic culture of hESCs from the previous dataset, hypermethylation at the CHCHD2 promoter was evident in P4 hESCs, as determined by the reactivity to McrBC, the methyl-specific DNA nuclease (Fig. 2H). In addition, tri-methylation of histone 3 lysine 9 (H3K9me3), typical histone modifications associated with gene repression [43], was increased, while acetylation of histone 3 lysine 27 (H3K27Ac) was markedly diminished in *CHCHD2* promoter region in P4 hESCs (Fig. 2 I). *CHCHD2* repression through repetitive in vitro culture (with enzymatic dissociation) was also apparent in another hESCs line (CHA3-hESCs) [44] (Fig. S2A and B) and iPSCs (BJ-iPSCs) with different passage numbers (EP: 67 CHA3-hESC passages, 35 BJ-iPSC passages; LP: 328 CHA3-hESC passages, 165 BJ-iPSCs passages] (Fig. S2C and D).

### *CHCHD2* knockout hESC establishment and initial characterization

**Fig. 3:**
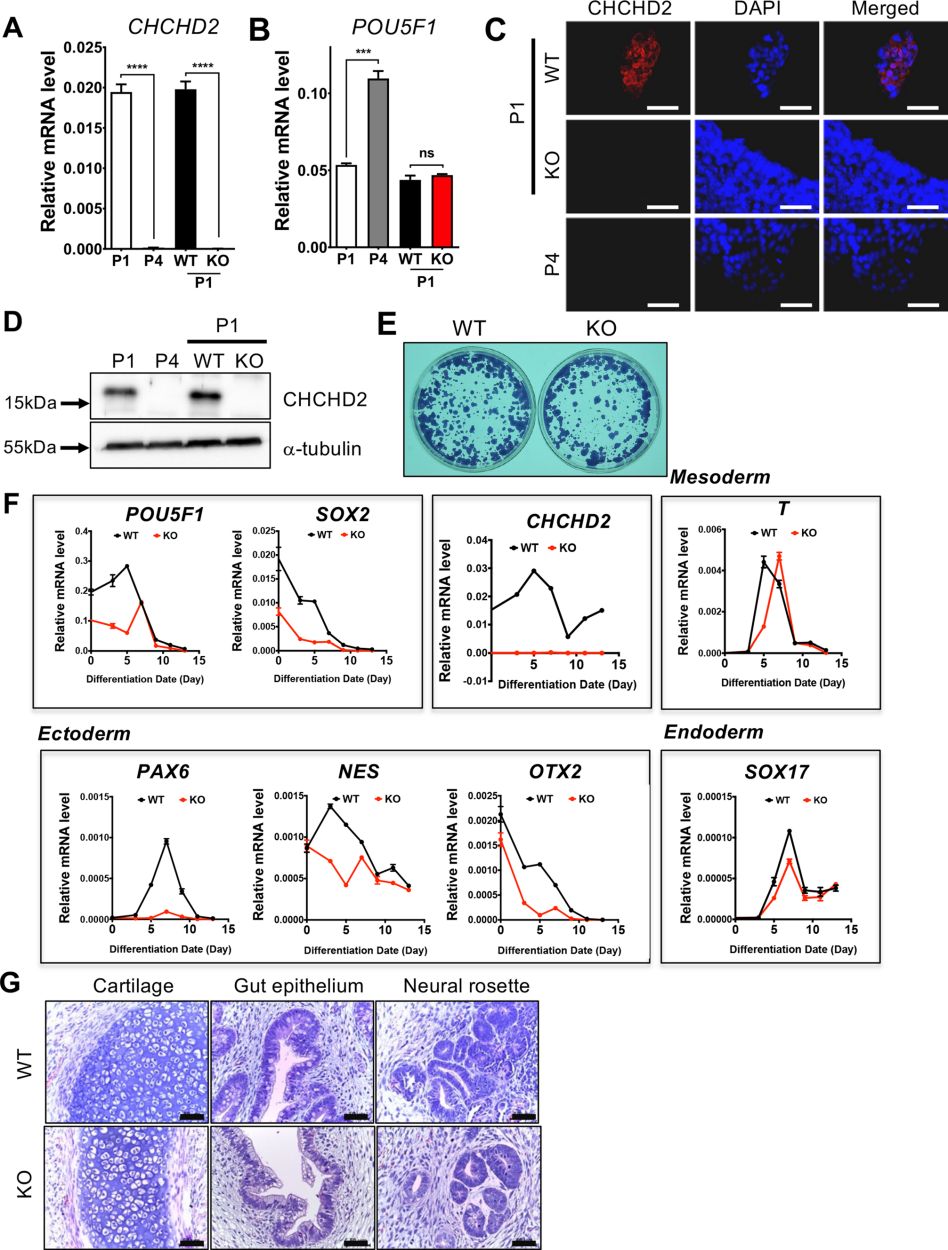
Establishment of *CHCHD2* knockout hESCs with CRISPR/Cas9 (**A**, **B**). **A** mRNA expression of *CHCHD2* in normal early passage (P1), long-term cultured (P4), *CHCHD2* WT, and KO hESCs, **B** mRNA expression of *POU5F1* for pluripotent gene marker (*n* = 2). **C** Immunofluorescent assay for *CHCHD2* expression, DAPI for nucleus staining (scale bar = 50 μm). **D** Immunoblot assay for *CHCHD2* protein expression, α-tubulin for equal protein loading control. **E** Alkaline phosphatase activity assay in WT and *CHCHD2* KO-hESCs. **F** mRNA expression during in vitro spontaneous differentiation of WT and *CHCHD2* KO-hESCs, *POU5F1,* and *SOX2* for pluripotent gene marker (*n* = 2). **G** Teratoma formation for confirmed three-germ layer differentiation in WT and *CHCHD2* KO-hESCs (scale bar = 50 μm)

Next, we established a *CHCHD2* knockout (KO) model in P1 hESCs (i.e., normal) using the previously described CRISPR/Cas9 technique [45] to monitor biological consequences from *CHCHD2* repression during hESCs enzymatic dissociation culture. Two sgRNA targeting sequences at introns 2 and 3 were designed to excise *CHCHD2’s* exon 3 (Fig. S3A). Simple genotyping analysis (Fig. S3B) and Sanger sequencing demonstrated the single clone establishment, lacking *CHCHD2’s* exon 3 in P1 hESCs at two alleles (hereafter KO hESCs) (Fig. S3C). Unlike the KO approach using CRISPR/Cas9 with one sgRNA to induce frame-shift after indel (insertion and deletion) formation, exon three deletion for *CHCHD2* KO allowed us to verify complete *CHCHD2* KO through simple RT-PCR analysis as it is similar to P4 hESCs’ level (Fig. 3A).

Of note, the *POU5F1* level in KO hESCs was comparable to the parent P1 hESCs (Fig. 3B). Immunostaining and immunoblotting analysis confirmed that complete mitochondrial *CHCHD2* protein loss in KO hESCs was similar to that of P4 hESCs (Fig. 3C and D). The initial KO hESCs pluripotency characterization was determined through key pluripotent mRNA level markers (data not shown) and the comparable colony level with alkaline phosphatase activity (Fig. 3E). One study has demonstrated that *CHCHD2* expression primes neuroectodermal differentiation by sequestering SMAD4 at mitochondria through direct protein binding [37]. Consistently, clear ‘TGFβ receptor signaling’ enrichment in the WikiPathway (Fig. S3D) and *ID1* induction, a common TGFβ downstream gene, in KO hESCs revealed functional *CHCHD2* KO was achieved (Fig. S3E). Accordingly, *PAX6*, a typical neuroectodermal determinant [46], was markedly attenuated in KO hESCs during spontaneous differentiation, while comparable level of mesoderm, endoderm, and pluripotency markers in KO hESCs (Fig. 3F). These data imply that *CHCHD2* loss solely impairs neuroectodermal differentiation, as previously described [37]. However, KO hESCs-derived teratoma also developed a neural rosette structure similar to WT hESCs, and no distinct alteration was observed in the three germ layers (Fig. 3G), which was consistent with the normal development of *CHCHD2* KO mice [47]. Additionally, we noted that hallmark ‘Apoptosis’ was associated with KO hESCs (Fig. S3F). In particular, there is a report that mitochondrial *CHCHD2* interacts with Bcl-xL to inhibit mitochondrial apoptosis in a cancer cell line model [35].

### Survival trait acquisition from *CHCHD2* loss

**Fig. 4:**
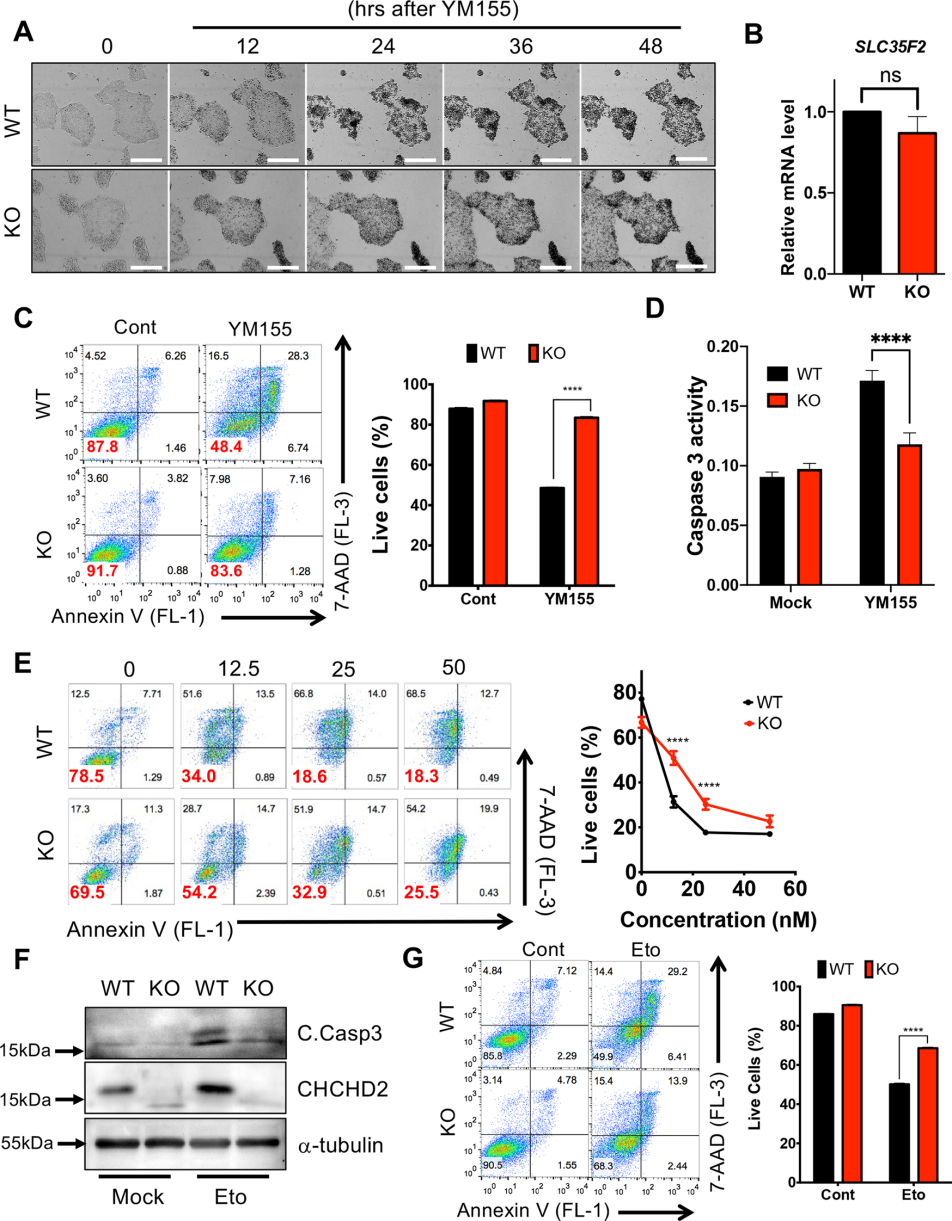

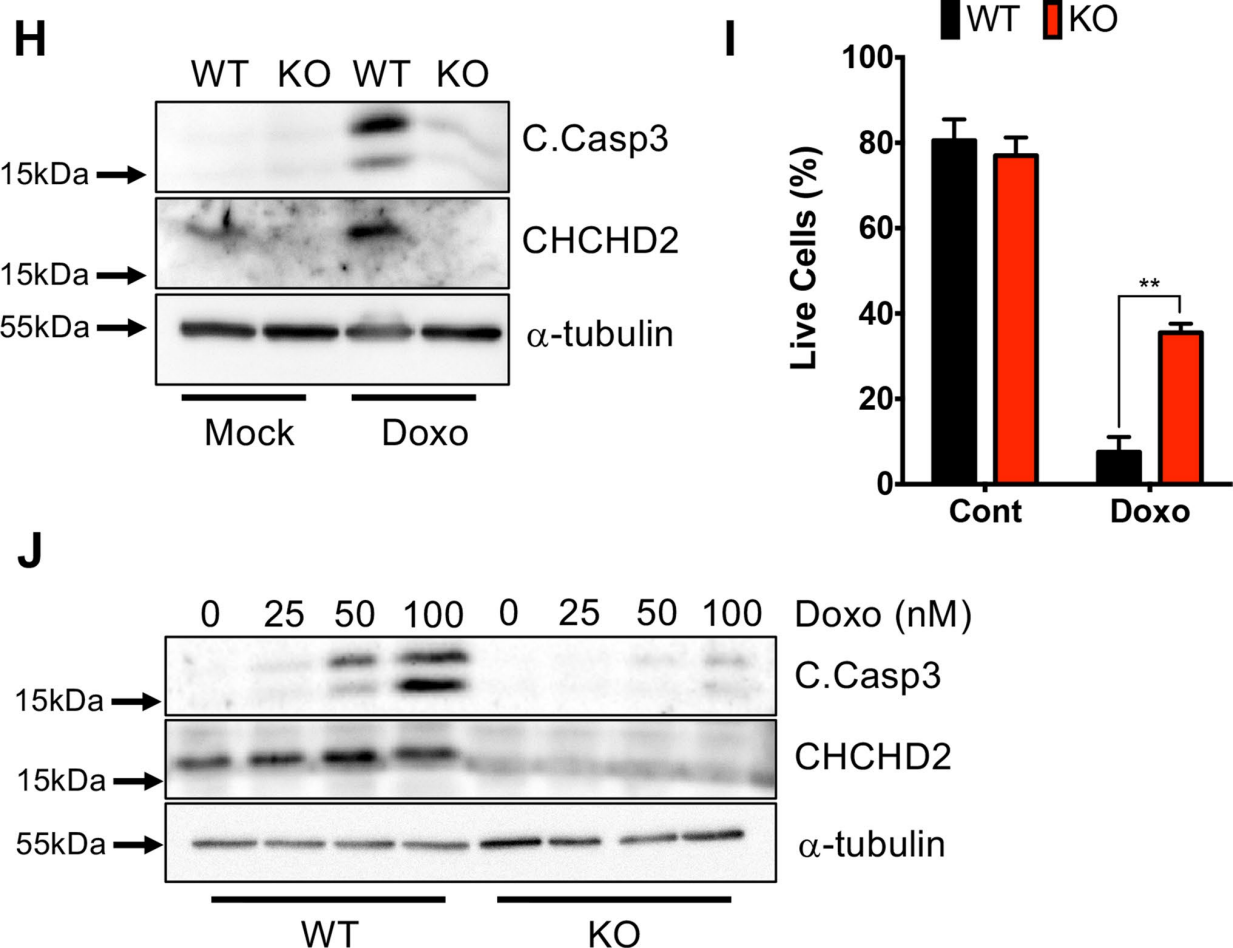
Acquire resistance of genotoxic stress in *CHCHD2* KO-hESCs. **A** Time-dependent optical microscopic image upon 10 nM of YM155 in WT and *CHCHD2* KO-hESCs (Scale bar = 500 μm). **B** mRNA expression of SLC35F2 in WT and *CHCHD2* KO-hESCs (*n* = 2). **C** Flow cytometry analysis (left) and bar graph for quantification (right) with 10 nM of YM155 in WT and *CHCHD2* KO-hESCs (*n* = 3). **D** Graphical presentation of caspase-3 activity at 24 h after treatment of 10 nM of YM155 in WT and *CHCHD2* KO-hESCs (*n* = 3). **E** Flow cytometry for Annexin V/7-AAD analysis (left) and quantification graph (right) of WT and *CHCHD2* KO-hESCs in YM155-dependent manner (*n* = 2). **F** Immunoblot assay with 50 nM of etoposide (Eto) in WT and *CHCHD2* KO-hESCs, and α-tubulin for equal protein loading control. (G) Flow cytometry for Annexin V/7-AAD analysis (left) and quantification graph (right) of WT and CHCHD2 KO-hESCs in 100 nM of etoposide (*n* = 2). **H** Immunoblot assay with 50 nM of doxorubicin (Doxo) in WT and *CHCHD2* KO-hESCs, and α-tubulin for equal protein loading control. (I) Quantification graph for trypan blue assay of WT and CHCHD2 KO-hESCs in 50 nM of doxorubicin (*n* = 2). **J** Immunoblot assay of WT and *CHCHD2* KO-hESCs in Doxo-dependent manner, and α-tubulin for equal protein loading control

According to the study that CHCHD2 inhibits mitochondrial apoptosis [35], we examined whether KO hESCs were more susceptible to mitochondrial apoptosis than WT. To trigger hESC cell death, we utilized YM155, a small molecule, inducing the selective cell death of undifferentiated hPSCs [7] via selective SLC35F2 cellular import, a solute carrier protein, highly expressed in hPSCs [45, 48]. Unexpectedly, KO hESCs were rather less sensitive to YM155 than WT (Fig. 4A and Movie S1) despite their comparable SLC35F2 expression levels (Fig. 4B). KO’s unexpected YM155 resistance was confirmed through flow cytometry analysis (Fig. 4C), caspase activity assay (Fig. 4D), and a YM155 dose-dependent challenge (Fig. 4E). KO hESCs were also more resistant to genotoxic agents such as etoposide (Eto, Fig. 4F and G) and doxorubicin to the parent control (Doxo, Fig. 4H and I). The resistance of KO hESCs to doxorubicin was highlighted by apoptosis level in a dose-dependent manner (Fig. 4J). Notably, resistance to these genotoxic agents was observed in culture-adapted hESCs (P3 and P4 hESCs), where *BCL2L1* was highly induced [20]. These data imply that *CHCHD2* loss in hESCs through repetitive enzymatic dissociation culture (Figs. 1 and 2) favors survival.

### *CHCHD2* loss rescues hESCs from dissociation-induced cell death

Culture-adapted hESCs survive diverse stresses derived from culture conditions (e.g., dissociation-induced apoptosis from enzymatic dissociation) [49] by inducting *BCL2L1*, a common factor for ‘culture adaptations’ [18] and TP53 mutations [22]. Thus, aberrant clones eventually become dominant through ‘winning’ the competition. As *CHCHD2* repression was the most evident in hESCs late passage through the ‘enzymatic dissociation’ culture method (Fig. 1G), we surmised that *CHCHD2* repression is an adapted response from ‘enzymatic dissociation’ and would favor survival upon dissociation-induced apoptosis.

**Fig. 5:**
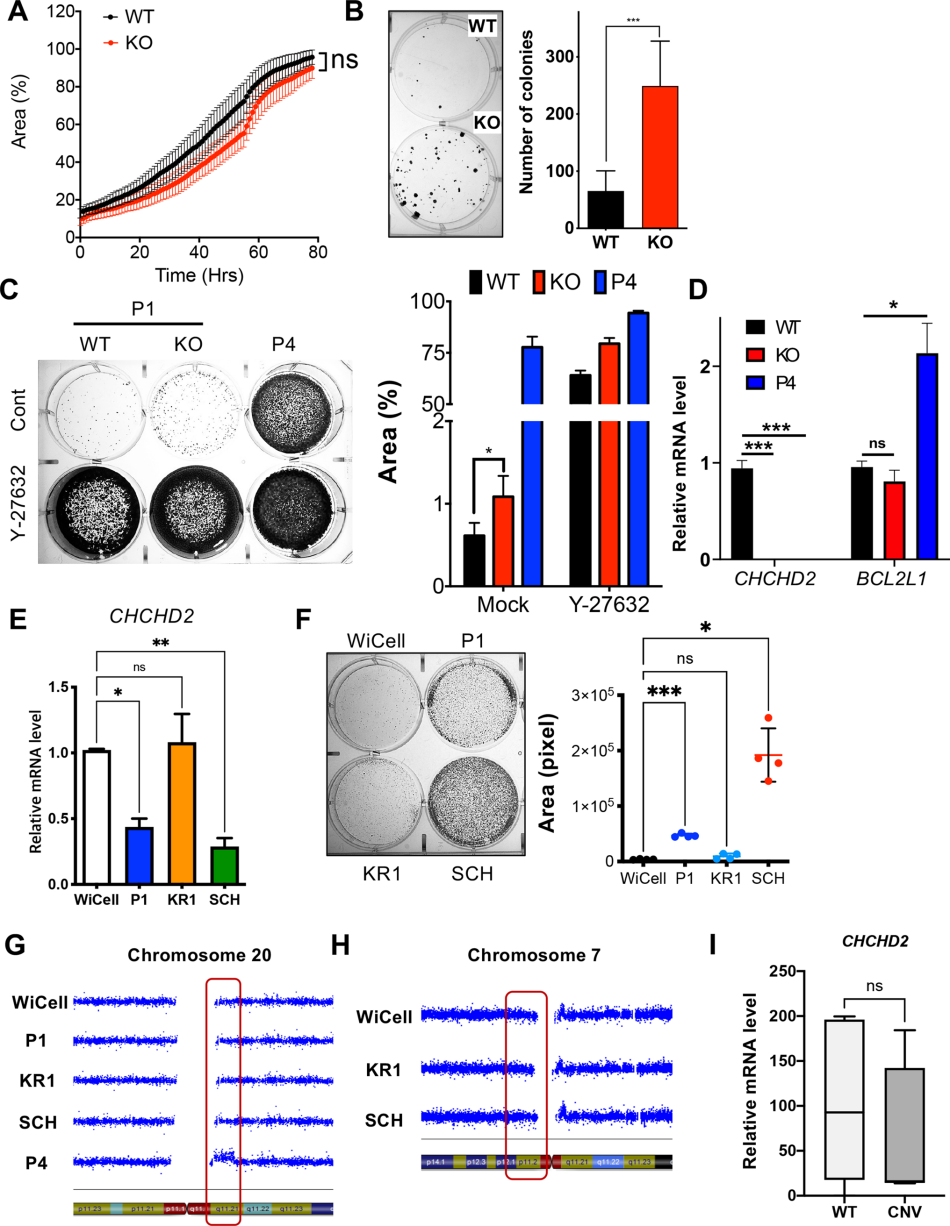
Acquire survival trait under single cell dissociation-induced cell death by wane of *CHCHD2*. **A** Growth curve of WT and *CHCHD2* KO-hESCs for 72 h (*n* ≥ 3). **B** Image of clonogenic assay (left) and quantification bar graph (right) of single cell-dissociated WT, *CHCHD2* KO-hESCs without ROCK inhibitor (Y-27632) (*n* = 3). **C** Image of clonogenic assay (left) and quantification bar graph (right) of single cell-dissociated WT and *CHCHD2* KO hESCs with or without Y-27632, and late passage (P4) hESCs for positive single cell-dissociated stress resistance control (*n* = 4). **D** mRNA expression of *CHCHD2* and *BCL2L1* in WT, *CHCHD2* KO, and late passage (P4) hESCs (*n* = 2). **E** mRNA expression of *CHCHD2* in H9 variants with different culture methods. **F** Image of clonogenic assay (left) and quantification graph (right) of single cell-dissociated H9 variants with different culture methods. **G** and **H** Log R ratio (LRR) plot of H9 sublines for chromosome 20 (**G**) and chromosome 7 (**H**) indicating the relative abundance of the genomic DNA around the SNP, which is expected to correlate with the copy number. **I** mRNA expression level of *CHCHD2* in WT and CNV (in 20q11.21 loci) occurred hiPSCs (*n* ≥ 4)

Despite comparable growth rate of KO compared to WT (Fig. 5A and Movie S2), KO hESCs formed more colonies after enzymatic dissociation without Y-27632 supplementation (to mimic a dissociation-induced apoptosis condition) (Fig. 5B). The survival difference between WT and KO hESCs disappeared with Y-27632 treatment (Fig. 5C). Of note, culture-adapted hESCs with distinct CNV at 20q11.21 (P4 hESCs) retained high *BCL2L1* expressions [20] and YAP activity [25], showing a more distinct survival phenotype than *CHCHD2* loss under the same conditions (Fig. 5C). However, KO hESCs expressed a comparable level of *BCL2L1* unlike that of P4 hESCs (Fig. 5D, S4A and B). Thus, it is intriguing that KO hESCs independently survived from single cell dissociation-induced apoptosis in *BCL2L1*.

Since ROCK inhibitor (Y-27632) rescues hPSCs’ specific dissociation-induced apoptosis [42], which has been a considerable hurdle for routine cell culture, Y-27632 was widely used to improve cell viability during hPSC culture [50]. Thus, other than prompt metabolic change by transient exposure [51] and change in actin filament by prolonged exposure [52], we surmise that repetitive exposure of Y-27632 would affect CHCHD expression. To this end, we collected three additional H9 hESCs from two independent institutes in Korea, which have been maintained under different culture protocols (Fig. S4C). *CHCHD2* expression levels from P1 hESCs experiencing 15 passages with enzymatic dissociation (55 passages, hereafter P1) from the original stock (40 passage), hESCs from Soonchunhyang university (hereafter SCH), and hESCs from Korea Research Institue of Bioscience and Biotechnology (KRIBB, hereafter KR1) maintained in different culture conditions (Fig. S4C) were examined and compared to H9 hESCs expanded from the original WiCell stock (WiCell, passage number 28) by one passage under our culture condition. Consistently, KR1 hESCs maintained in the feeder without Y-27632 supplementation (Fig. S4C) kept *CHCHD2* levels similar to WiCell hESCs (Fig. 5E). In parallel, KR1 hESCs and WiCell expressed a more distinct sensitivity to dissociation-induced apoptosis, unlike other hESCs (Fig. 5F).

One study elucidated *CHCHD2* downregulation as a 20q11.21 gain marker [53]. However, the set of H9 hESCs with different expression level of CHCHD2 (Fig. 5E) were all normal copy numbers in 20q11.21 (Fig. 5G) as well as 7p11.2 (Figs. 2G and 5H) where *CHCHD2* gene is located. Additionally, there was no noticeable correlation of *CHCHD2* expression to 20q11.21 gain in nine iPSCs (5 iPSCs with normal copy number: WT; 4 iPSCs with 20q11.21 gain: CNV) maintained without Y-27632 supplement (under 35 passages) (Figs. 5I and S4D).

### *CHCHD2* expression for cell death susceptibility

**Fig. 6:**
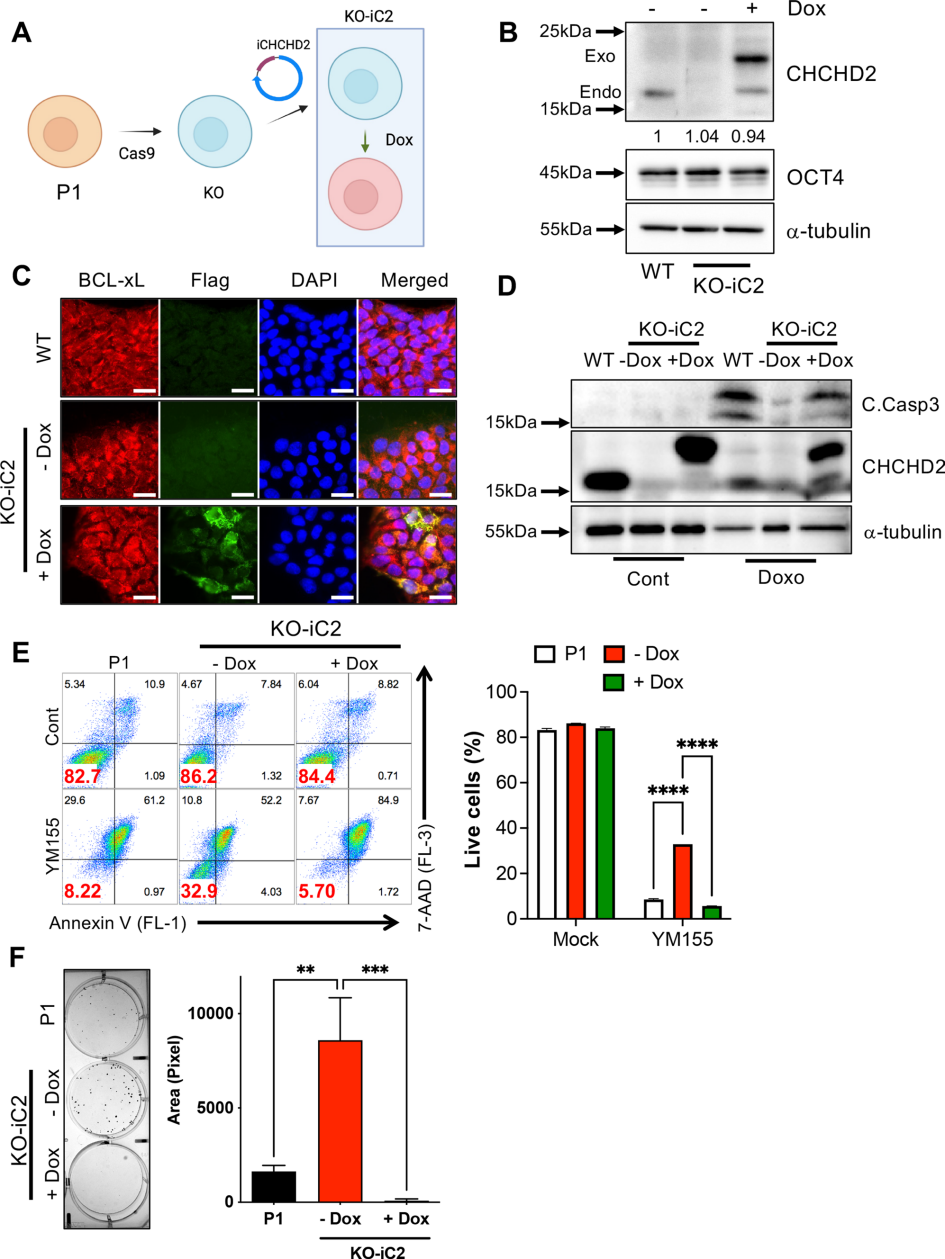
Reverted survival trait by reconstitution of *CHCHD2* in hESCs. **A** Schematic images for construction of doxycycline-inducible *CHCHD2* reconstitution (iC2) cell model in *CHCHD2* KO-hESCs. **B** Immunoblot assay of WT, *CHCHD2* KO, and iC2 hESCs with 0.25 μg/mL of Dox, and α-tubulin for equal protein loading control. **C** Immunofluorescent assay for BCL-xL and *CHCHD2*-Flag in WT and iC2 hESCs with 0.1 μg/mL Dox, DAPI for nucleus staining (Scale bar = 25 μm). **D** Immunoblot assay of WT, KO, and iC2 hESCs with 40 nM of Doxo, and α-tubulin for equal protein loading control. **E** Flow cytometry for Annexin V/7-AAD analysis (left) and quantification bar graph (right) with 10 nM of YM155 in WT, *CHCHD2* KO, and iC2 hESCs (*n* = 2). **F** Optical microscopic images of clonogenic assay (left) and quantification bar graph (right) of single cell-dissociated early passage (P1) and *CHCHD2* reconstitution hESCs with or without 0.25 μg/mL Dox without ROCK inhibitor (*n* = 5)

Next, we confirmed *CHCHD2*’s role in cell death susceptibility by producing doxycycline (Dox) inducible *CHCHD2* in KO hESCs (KO-iC2) (Fig. 6A). *POU5F1* expression levels were not significantly altered regardless of *CHCHD2* expression (Fig. S5A). Simple Dox treatment markedly increased CHCHD2 levels with no distinct change of OCT4 protein levels (Fig. 6B). Expressed mitochondrial CHCHD2 (with FLAG-tag) was further validated through immunofluorescence with FLAG antibody (Fig. 6C). As predicted, *CHCHD2* reconstitution in KO-hESCs re-sensitized KO hESCs to Doxo- (Fig. 6D) and YM155-induced cell death (Fig. 6E and S5B) without significant *BCL2L1* and *SLC35F2* alteration, respectively (Fig. S5C). Survived colonies after single cell dissociation in KO-iC2 were markedly reduced by *CHCHD2* reconstitution with Dox (Fig. 6F).

### *CHCHD2* expression for ROCK activity

**Fig. 7:**
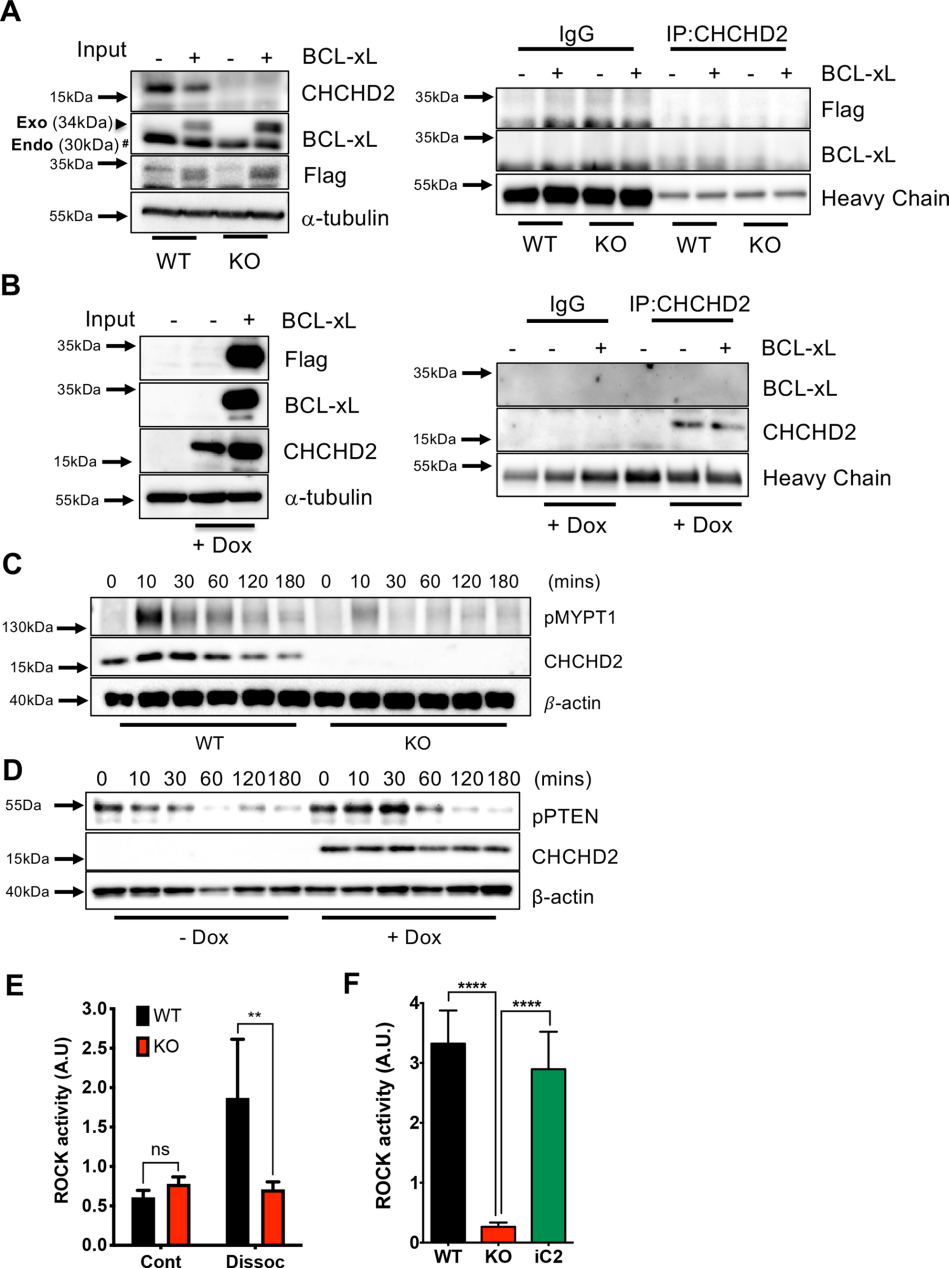
*CHCHD2* regulates ROCK activity. **A** Immunoprecipitation assay of WT and *CHCHD2* KO-hESCs with exogeneous expression of Bcl-xL-Flag, heavy chain for equal primary antibody control and α-tubulin for equal protein loading control. **B** Immunoprecipitation assay of iC2 hESCs with exogeneous expression of Bcl-xL-Flag. **C** Immunoblot assay of WT and *CHCHD2* KO-hESCs with incubation after single cell dissociation, and β-actin for equal protein loading control. **D** Immunoblot assay of *CHCHD2* KO and iC2 hESCs with incubation after single cell dissociation, and β-actin for equal protein loading control. **E** Kinase activity assay for ROCK2 kinase in single cell dissociated WT and *CHCHD2* KO hESCs with 0.25 μg/mL of Dox (*n* = 4). **F** Kinase activity assay for ROCK2 kinase in single cell dissociated WT, *CHCHD2* KO, and iC2 hESCs with 0.25 μg/mL of Dox (*n* = 4)

We were surprised that *CHCHD2* loss favored survival under diverse culture stresses in hESCs (Figs. 4 and 5) because CHCHD2 was previously determined to inhibit apoptosis through direct BCL-xL interaction in cancer cell lines [35]. First, we examined whether CHCHD2 interacts with BCL-xL in hESCs. BCL-xL (with FLAG tag) was ectopically expressed in WT and KO hESCs, and co-immunoprecipitation with CHCHD2 was carried out to emphasize this interaction. Unexpectedly, neither ectopically (Exo) expressed nor endogenous (Endo) BCL-xL was pulled down alongside CHCHD2 immunoprecipitation (Fig. 7A). Additionally, CHCHD2 co-immunoprecipitation repetition in KO-iC2 after Dox treatment also failed to draw BCL-xL out (Fig. 7B). Thus, we surmised that the lack of CHCHD2 and BCL-xL interaction in hESCs account for the contradictory CHCHD2 effect on apoptosis in hESCs.

Repetitive enzymatic dissociation was likely to repress *CHCHD2* (Fig. 1) and favor survival from dissociation-induced apoptosis. Thus, we surmised that *CHCHD2* expression affected ROCK activity, a primary cell death determinant, of which attenuation occurs in culture adapted hPSCs [54]. To this end, phosphorylated myosin phosphatase target subunit 1 (MYPT1), a direct ROCK substrate, [55] levels were monitored after single cell dissociation. MYPT1 phosphorylated 10 min after single cell dissociation was markedly diminished in KO hESCs, suggesting that CHCHD2 expression contributes to ROCK activation (Fig. 7C). Consistently, ROCK-regulated PTEN phosphorylation levels [56] notably increased through CHCHD2 reconstitution in KO-iC2 hESCs after single cell dissociation (Fig. 7D). Additionally, phosphorylation of cofilin (pCofilin), occurring in ROCK activity-dependent manner through LIM-kinase activation [57], was also noticeably decreased in KO hESCs upon single cell dissociation (Fig. S6A). The level of pCofilin upon dissociation was recovered by reconstitution of CHCHD2 expression (Fig. S6B). A biochemical assay quantified ROCK activity after single cell dissociation to validate these results. ROCK activity drastically increased immediately after cell dissociation and remained elevated for 180 min (Fig. S6C). Maximum ROCK activity (30 min after dissociation) was markedly attenuated in KO hESCs (Fig. 7E) and was regained after CHCHD2 reconstitution in KO-iC2 hESCs (Fig. 7F). Unlike cancer cells, where ROCK activity governs actin organization to promote cell migration, the level of CHCHD2 expression gave only marginal effect on migration capacity (Fig. S6D and E). These data conclusively imply that *CHCHD2* epigenetic repression during in vitro culture (through enzymatic dissociation in particular) is another cellular adaptive event to endow ‘survival traits,’ through repression of ROCK activity upon dissociation, leading to cellular dominancy like TP53 mutations [22], *BCL2L1* induction [18], or YAP activation [25].

## Discussion

Tissue regeneration through hPSCs-derived cell therapy has drawn substantial attention since the first human autologous stem cell therapy’s promising clinical outcome [58] and recent FDA approval for Parkinson’s disease hESC-based phase I trial (run by ‘BlueRock Therapeutics’). However, hPSC-based cell therapy safety has been a consistent societal concern due to the uncertainty of cellular and genomic profile alteration during in vitro culture [17]. ‘Survival trait’ acquisition during in vitro culture (or culture adaptation [14]) due to *BCL2L1* induction [18, 20, 53], *TP53* mutation [22], or YAP activation [25] leads to abnormal clonal dominance and exacerbates genetic aberrations by escaping abnormal mitosis [8]. We confirmed that epigenetic repression of *CHCHD2*, occurred specifically through repetitive ‘enzymatic dissociation’ culture by in silico analysis of methylome data [41] (Fig. 1H and I), McrBC reactivity and ChIP assay with an in-house model (Fig. 2H and I). This cellular event is associated with the acquisition of ‘survival trait’ through ROCK activation interference.

A previous study substantiated that *CHCHD2* loss, as a 20q11.21 gain marker, affects neuroectodermal differentiation [53]. Similarly, we observed that neuroectodermal lineage, determined by *PAX6* expression during spontaneous differentiation, was impaired in *CHCHD2* KO (Fig. 3F). P3 and P4 hESCs with apparent 20q11.21 gain completely repressed *CHCHD2* (Fig. 2) [25]. However, *CHCHD2* expression level in early passaged iPSCs varied regardless of 20q11.21 gain (Fig. 5I). Instead, *CHCHD2* levels in the relatively early passage of four different hESCs (collected from independent Korean institutes through different culture methods) (Figs. 5E and S4C) were consistent with transcriptome analyses from early and late hPSCs maintained with different culture conditions [41]. Accordingly, we suggest that *CHCHD2* repression would be a ‘repetitive enzymatic dissociation’ marker rather than gain of 20q11.21. Considering the complete *CHCHD2* repression, possibly due to closed chromatin accessibility of *CHCHD2* promoter region (Fig. 2H and I) in P4 hESCs with a 20q11.21 gain and the previous study [53], we could not exclude the possibility that 20q11.21 gain in hPSCs could have resulted from the repetitive culture with ‘enzymatic dissociation’. This possibility requires additional follow-up studies.

Unlike previous cancer cell line studies demonstrating that mitochondrial *CHCHD2* interacts with BCL-xL to inhibit apoptosis [35], *CHCHD2* KO in hESCs inhibited mitochondrial cell death induced by genotoxic insults (Fig. 4), which was re-sensitized by *CHCHD2* reconstitution (Fig. 6). Despite multiple attempts, CHCHD2 in BCL-xL interactions could not be reproduced in hESCs (Fig. 7A and B), implying that CHCHD2’s role in apoptosis is distinct in hESCs. We also tested whether *CHCHD2* reconstitution in P4 hESCs with clear 20q11.21, consequent *BCL2L1* induction, and YAP activation would sensitize cell death induced by ‘single cell dissociation’ or genotoxic stress. As high BCL-xL expression, an anti-apoptotic protein, desensitizes ‘mitochondrial apoptosis’ priming, it endows ‘the strong selective advantage’ [18] in P4 hESCs; *CHCHD2* expression failed to overcome the survival trait (data not shown). These data imply that high BCL-xL expression or YAP activity is major determinant for ‘survival advantage’ in hESCs.

Given the lack of an early marker for ‘survival trait acquisition’ or ‘culture adaptation’ cellular events other than *BCL2L1* induction or CNV at 20q11.21, *CHCHD2* expression level may be used as a ‘repetitive culture with enzymatic dissociation’ and ‘survival trait’ from ‘dissociation induced cell death’ indicator useful in routine hPSCs assessments.

### Supplementary Information

Below is the link to the electronic supplementary material.

Supplementary file1 (PDF 2351 KB)

Supplementary file2 (MP4 9320 KB)

Supplementary file3 (MP4 8966 KB)

Supplementary file4 (MP4 7340 KB)

Supplementary file5 (MP4 11321 KB)

## Data Availability

Source data are available from the *Cellular and Molecular Life Sciences* online or corresponding authors upon request.

## Abbreviations

hPSCs: Human pluripotent stem cells
hESCs: Human embryonic stem cells
iPSCs: Induced pluripotent stem cells
CHCHD2: Coiled-coil-helix-coiled-coil-helix domain containing 2
CNV: Copy number variation
EP: Early passage
LP: Late passage
Me: Mechanical
En: Enzymatic
DMC: Differentially methylated CpG
DMRs: Differentially methylated regions
ROCK: Rho-associated protein kinase
WT: Wild type
KO: Knockout
RT-PCR: Real-time polymerase chain reaction
Dox: Doxycycline
Doxo: Doxorubicin
Eto: Etoposide
Endo: Endogenous
Exo: Ectopically
ChIP: Chromatine immunoprecipitation
