# Supplementary material for: Epigenetic repression of CHCHD2 enhances survival from single cell dissociation through attenuated Rho A kinase activity
Source: Cell Mol Life Sci. 2024 Jan 12;81(1):38. doi: 10.1007/s00018-023-05060-8 (PMC10787008; doi:10.1007/s00018-023-05060-8)
Supplement: Supplementary file 1 — Supplementary file1 (PDF 2351 KB) [file 18_2023_5060_MOESM1_ESM.pdf]

Supplementary Information

Supplementary Materials and Methods

Immunoblotting

Immunoblotting and immunofluorescent assay were performed as described previously. Antibody for *CHCHD2* (#19424-1-AP) was purchased from proteintech. Antibodies for VDAC1 (#4866) was purchased from Cell Signaling Technology. Antibodies for α-tubulin (#sc-5286) and β-actin (#sc-47778) were purchased from Santa Cruz Biotechnology. Antibody for BCL-xL (#ab32370).

Mitochondrial isolation

Mitochondrial isolations from human embryonic stem cells were performed by mitochondria isolation kit for cultured cells (ThermoFisher, #89874) according to the manufacturer's instructions. Mitochondria lysis for immunoblot were preserved by RIPA buffer or 2% CHAPS in TBS as described elsewhere.

ROCK activity assay

For analyzing ROCK activity assay, hESCs were detatched single cell by accutase (BD Bioscience, #561527) in 37°C CO_2 incubator for 3min, and also incubated time-dependent or single time point (30min) without ROCK inhibitor. Cells were washed PBS with twice and lysis by TLB. After lysis, the lysates were quantified by BCA analysis (ThermoFisher, #23227) in accordance with manufacturer's protocol. 100μg of total lysate was used for measuring ROCK activity by ROCK activity assay kit (Cell Biolabs, #STA-416) in accordance with the manufacturer's instruction.

Wound healing assay

For wound healing assay, hESCs were cultured in 6-wall culture plate with Matrigel coating, and proliferated up to 90% of cell density. Cells were wounded by 1mm scar scratcher (SPL, #201907) and live imaging by JuLI-Stage for 28hrs. The wound healing results was analyzed by JuLI-STAT software.

Supplementary Figure legends

Figure S1. (A) Table for hESCs with different culture matrices and methods. (B) The number of differentially methylated (DM) probes comparing high and low passage samples with different culture methods. (C) The log-fold change values and p-values of high passage samples compared to low passage samples were displayed by volcano plot. Areas with no significant methylation were illustrated in grey, while the different positions of each probe were represented by colors. (D) Venn diagram of up- and downregulated methylation probes in different culture methods.

Figure S2. (A) mRNA expression of *CHCHD2* in early (EP) and trisomy 12 late passage (LP) CHA3 hESCs. (B) Immunoblot assay for *CHCHD2* in EP and LP CHA3 hESCs, β-actin for equal protein loading control. (C) mRNA expression of *CHCHD2* in early (EP) and late passage (LP) BJ-iPSCs. (D) Immunoblot assay for *CHCHD2* in EP and LP BJ-iPSC, α-tubulin for equal protein loading control.

Figure S3. (A) Scheme for knockout strategy of *CHCHD2* in hESCs. (B) Genotyping analysis of sgRNA treated hESCs pool and six single clones. (C) Sanger sequencing data for *CHCHD2* wild-type (WT), and knockout (KO)-hESCs. (D-E) GO analysis with 'WikiPathways' of WT and *CHCHD2* KO-hESCs, and (E) mRNA expression of *ID1* for TGF-b downstream. *POU5F1* and *SOX2* for pluripotent gene marker ($n \geq 4$). (F) 'Hallmarks' of different enrichment genes (DEGs) of WT and *CHCHD2* KO-hESCs.

Figure S4. (A-B) Immunoblot assay of (A) early passage (P1) and late passage (P4), (B) WT and *CHCHD2* KO-hESCs with fractionation of cytosol and mitochondria, VDAC1 for equal mitochondria protein loading control and α-tubulin for equal cytosol protein loading control.

(C) Table for varied H9-hESCs with different passaging method from WiCell and two independent institute in Korea Republic. (D) Table for culture method of hiPSCs with CNV in 20q11.21 or not.

Figure S5. (A) (left) *POU5F1* mRNA expression of WT, *CHCHD2* KO, and *CHCHD2* reconstitution hESCs and (right) *CHCHD2* mRNA expression of *CHCHD2* reconstitution hESCs with 0.1 μm/mL Dox. (B) Flow-cytometry for Annexin V/7-AAD analysis (left) and quantification graph (right) of WT, *CHCHD2* KO, and KO-iC2 hESCs in YM155 dependent-manner (n=2). (C) mRNA expression of *BCL2L1* (left) and *SLC35F2* (right) of WT, *CHCHD2* KO, and *CHCHD2* reconstitution hESCs with 0.1 μm/mL Dox.

Figure S6. (A) Immunoblot assay of WT and *CHCHD2* KO-hESCs with incubation after single cell dissociation, α-tubulin for equal protein loading control. (B) Immunoblot assay of CHCHD2 KO-iC2 with or without 0.25 μg/mL with incubation after single cell dissociation, β-actin for equal protein loading control. (C) Kinase activity assay for ROCK2 kinase in single cell dissociated WT hESCs up to 180mins with 0.25 μg/mL of Dox (n=4). (D) Wound healing assay of WT and CHCHD2 KO-hESCs (n=5). (E) Wound healing assay of CHCHD2 KO-iC2 hESCs with or without 0.25 μg/mL of Dox (n≥5).

Movie S1 Live images of cell death of (A) WT and (B) CHCHD2 KO hESCs after YM155 treatment

Movie S2 Live images of cell growth of (A) WT and (B) CHCHD2 KO hESCs

Figure S1

A

Matrix	Dissociation	Passage	
Feeder	Mechanical	Early	P2
		Late	P103
	Enzyme (Accutase)	Early	P6
		Late	P104
ECM	Mechanical	Early	P6
		Late	P147
	Enzyme (Accutase)	Early	P5
		Late	P161

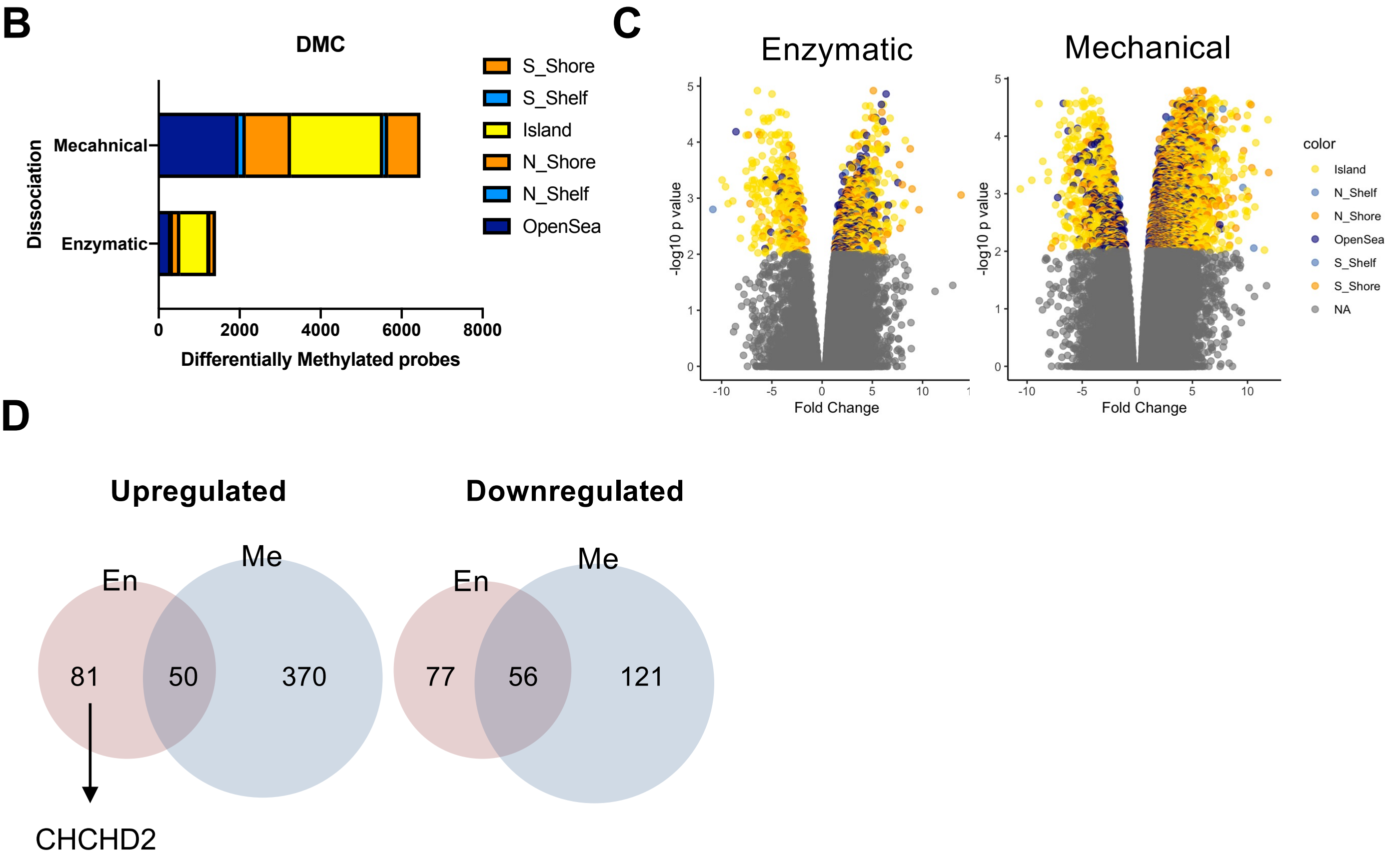

Figure S2

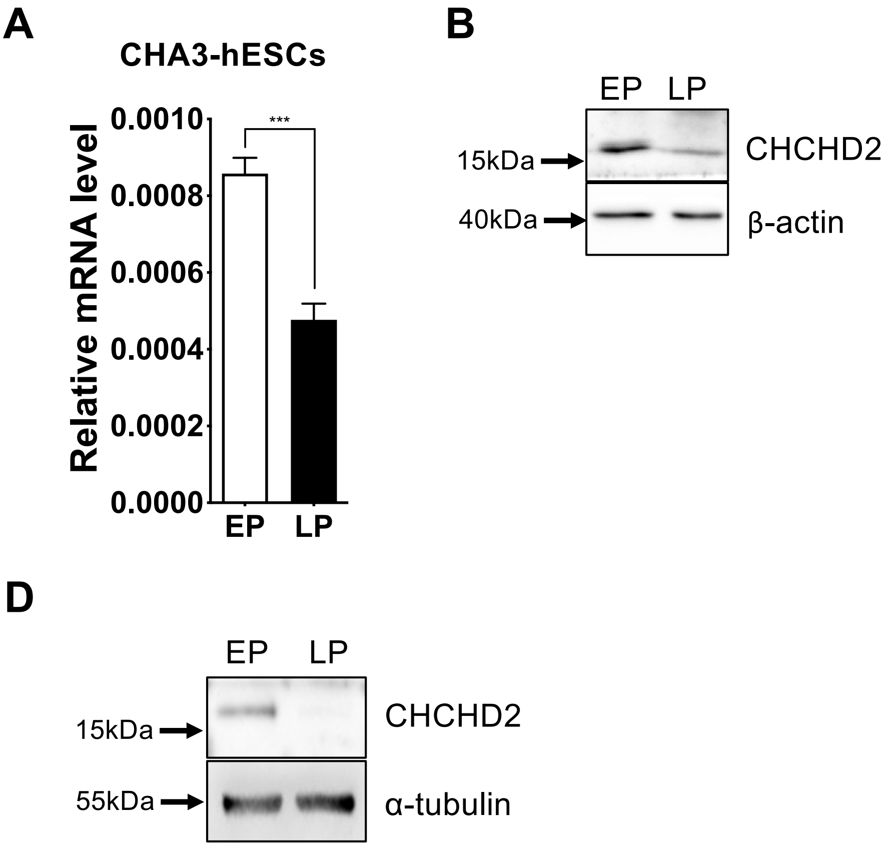
A
CHA3-hESCs
Relative mRNA level
0.0010
0.0008
0.0006
0.0004
0.0002
0.0000
***
EP
LP
B
EP
LP
15kDa
CHCHD2
40kDa
β-actin
C
BJ-iPSCs
Relative mRNA level
0.00005
0.00004
0.00003
0.00002
0.00001
0.00000
****
EP
LP
D
EP
LP
15kDa
CHCHD2
55kDa
α-tubulin

Figure S3

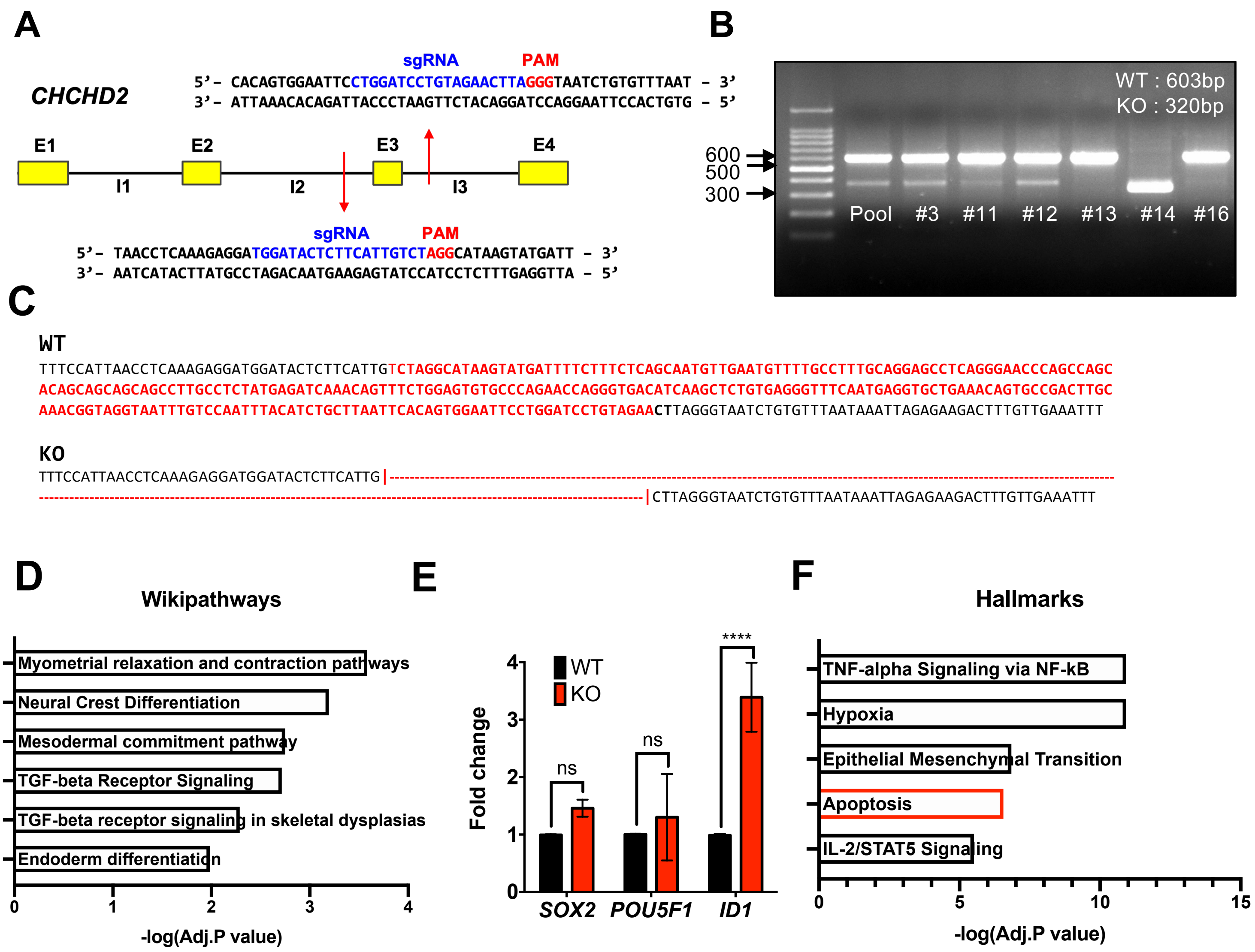

A
CHCHD2
sgRNA
PAM
5’– CACAGTGGAATTCCTGGATCCTGTAGAACTTAGGGTAATCTGTGTTTAAT – 3’
3’– ATTAAACACAGATTACCCTAAGTTCTACAGGATCCAGGAATTCCACTGTG – 5’
E1
E2
E3
E4
I1
I2
I3
sgRNA
PAM
5’– TAACCTCAAAGAGGATGGATACTCTTCATTGTCTAGGCATAAGTATGATT – 3’
3’– AATCATACTTATGCCTAGACAATGAAGAGTATCCATCCTCTTTGAGGTTA – 5’
B
WT : 603bp
KO : 320bp
600
500
300
Pool
#3
#11
#12
#13
#14
#16
C
WT
TTTCCATTAACCTCAAAGAGGATGGATACTCTTCATTGTCTAGGCATAAGTATGATTTTCTTTCTCAGCAATGTTGAATGTTTTGCCTTTGCAGGAGCCTCAGGGAACCCAGCCAGC
ACAGCAGCAGCAGCCTTGCCTCTATGAGATCAAACAGTTTCTGGAGTGTGCCCAGAACCAGGGTGACATCAAGCTCTGTGAGGGTTTCAATGAGGTGCTGAAACAGTGCCGACTTGC
AAACGGTAGGTAATTTGTCCAATTTACATCTGCTTAATTCACAGTGGAATTCCTGGATCCTGTAGAACTTAGGGTAATCTGTGTTTAATAAATTAGAGAAGACTTTGTTGAAATTT
KO
TTTCCATTAACCTCAAAGAGGATGGATACTCTTCATTG
CTTAGGGTAATCTGTGTTTAATAAATTAGAGAAGACTTTGTTGAAATTT
D
Wikipathways
Myometrial relaxation and contraction pathways
Neural Crest Differentiation
Mesodermal commitment pathway
TGF-beta Receptor Signaling
TGF-beta receptor signaling in skeletal dysplasias
Endoderm differentiation
0
1
2
3
4
-log(Adj.P value)
E
WT
KO
Fold change
0
1
2
3
4
ns
ns
****
SOX2
POU5F1
ID1
F
Hallmarks
TNF-alpha Signaling via NF-kB
Hypoxia
Epithelial Mesenchymal Transition
Apoptosis
IL-2/STAT5 Signaling
0
5
10
15
-log(Adj.P value)

Figure S4

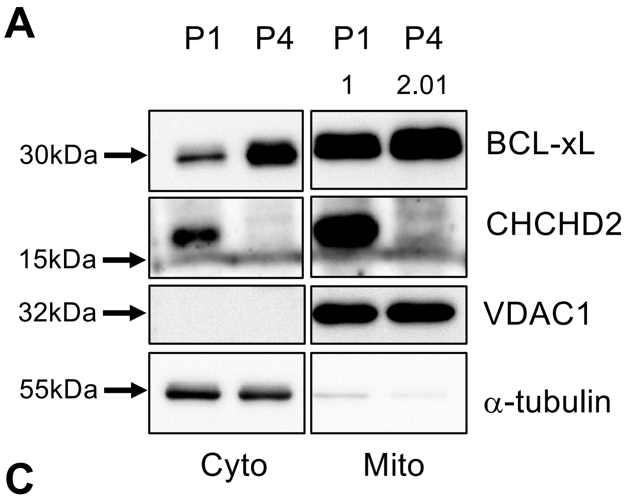

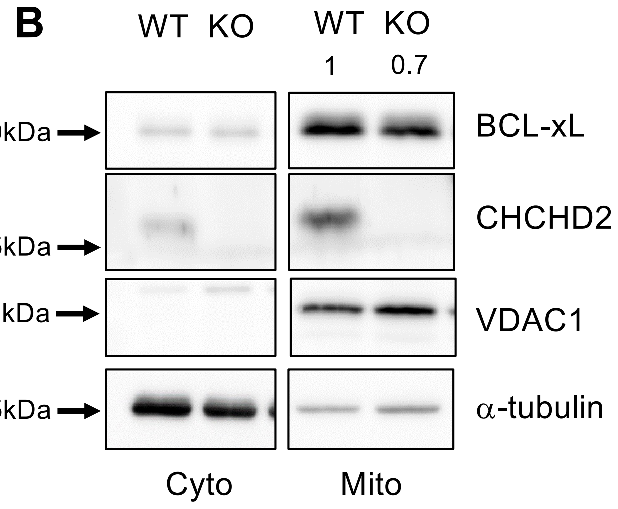

C

Institute	Cell Name	Matrix	Passaging Method	# passage	References (PMID)	Use of Y27632
WiCell	WiCell	Feeder	n.a	29	n.a	X
Seoul National University	P1	Matrigel	Dispase™ (Clump passaging)	55	30293852	O
KRIBB	KR1	Feeder	ReLeSR™ (Clump passaging)	44	34301945	X
Soonchunhyang University	SCH	Vitronectin	TrypLE™	81	32357563	O

D

Group	CN state (Chr20q11)	Institute	Cell Name	Matrix	Passaging Method	# passage	References (PMID)	Use of Y27632
WT	-	KNIH	KSCBi005-A	Vitronectin	EDTA	p20	33714852	X
WT	-	KNIH	CMC-hiPSC-006	Vitronectin	EDTA	p13	-	X
WT	-	KNIH	CMC-hiPSC-015	Vitronectin	EDTA	p15	-	X
WT	-	KNIH	CMC-hiPSC-016	Vitronectin	EDTA	p15	-	X
WT	-	KNIH	CMC-hiPSC-020	Vitronectin	EDTA	p12	-	X
CNV	O	KNIH	KSCBi002-A	Vitronectin	EDTA	p34	33714852	X
CNV	O	KNIH	KSCBi002-A	Vitronectin	EDTA	p34	33714852	X
CNV	O	KNIH	KSCBi002-A-1	Vitronectin	EDTA	p26	33714852	X
CNV	O	KNIH	CMC-hiPSC-018	Vitronectin	EDTA	p15	-	X

Figure S5

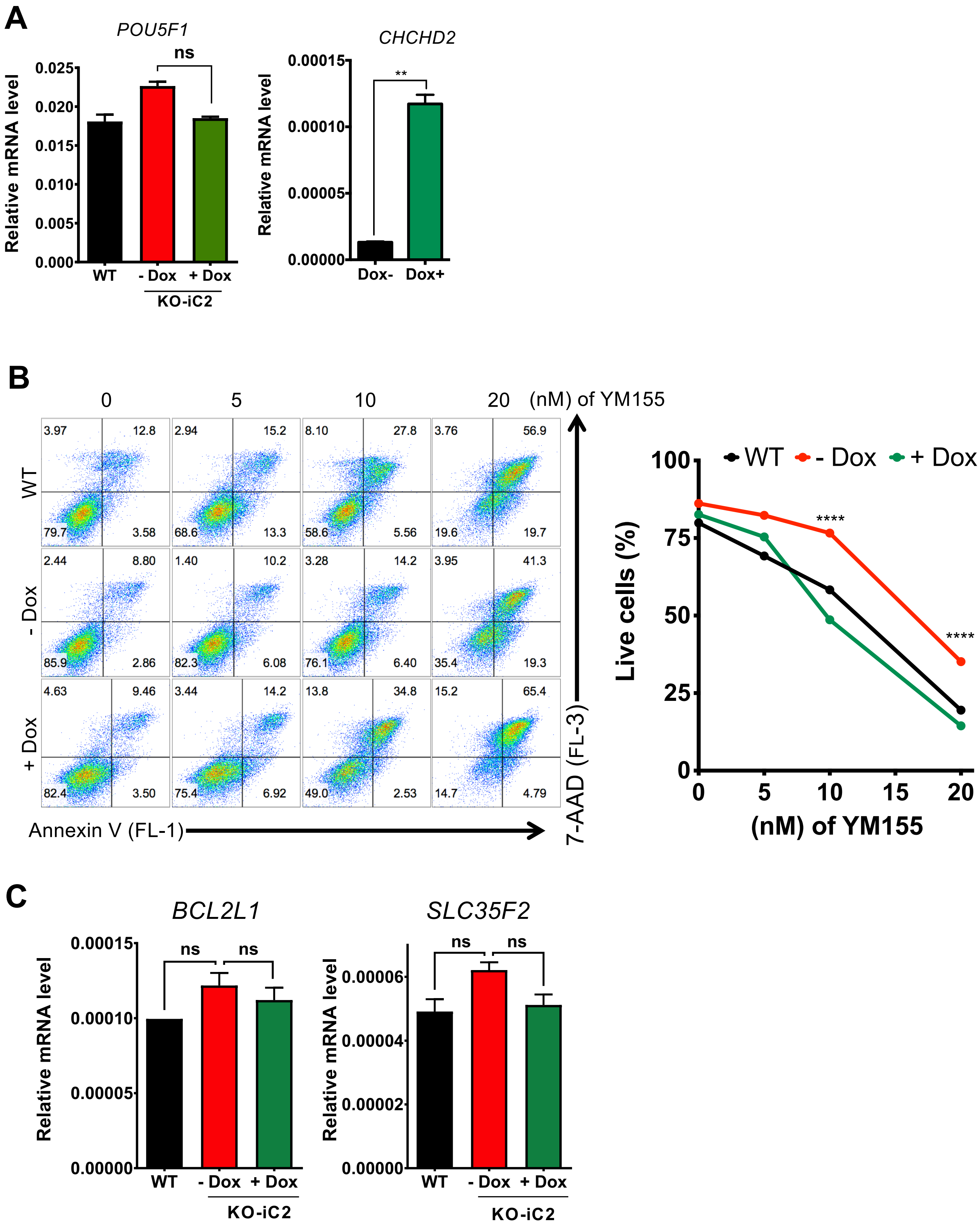

A
POU5F1
ns
Relative mRNA level
0.025
0.020
0.015
0.010
0.005
0.000
WT
- Dox
+ Dox
KO-iC2
CHCHD2
**
Relative mRNA level
0.00015
0.00010
0.00005
0.00000
Dox-
Dox+
B
0
5
10
20
(nM) of YM155
WT
- Dox
+ Dox
3.97
12.8
79.7
3.58
2.94
15.2
68.6
13.3
8.10
27.8
58.6
5.56
3.76
56.9
19.6
19.7
2.44
8.80
85.9
2.86
1.40
10.2
82.3
6.08
3.28
14.2
76.1
6.40
3.95
41.3
35.4
19.3
4.63
9.46
82.4
3.50
3.44
14.2
75.4
6.92
13.8
34.8
49.0
2.53
15.2
65.4
14.7
4.79
Annexin V (FL-1)
7-AAD (FL-3)
WT
- Dox
+ Dox
Live cells (%)
100
75
50
25
0
****
****
0
5
10
15
20
(nM) of YM155
C
BCL2L1
ns
ns
Relative mRNA level
0.00015
0.00010
0.00005
0.00000
WT
- Dox
+ Dox
KO-iC2
SLC35F2
ns
ns
Relative mRNA level
0.00006
0.00004
0.00002
0.00000
WT
- Dox
+ Dox
KO-iC2

Figure S6

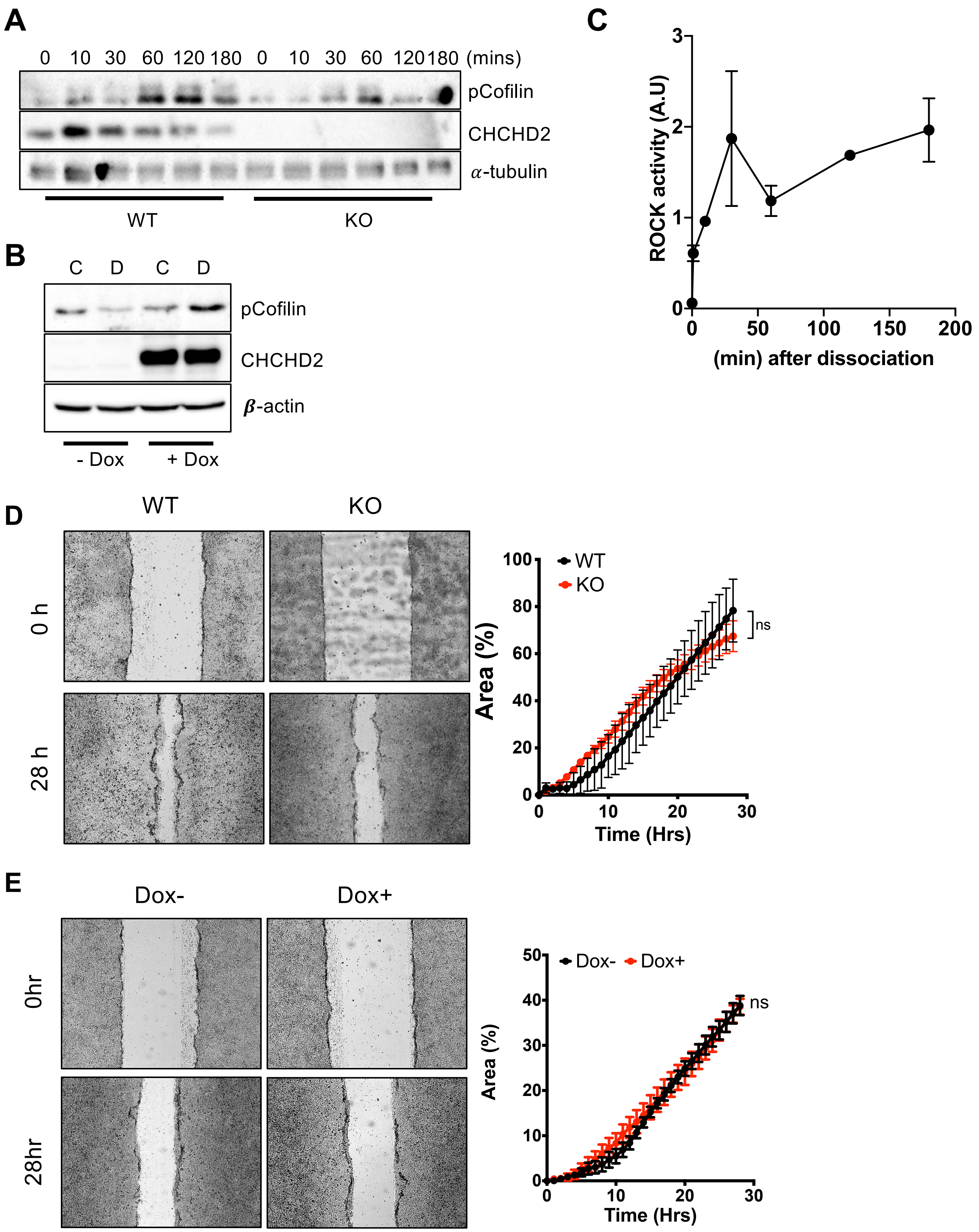
A
0 10 30 60 120 180 0 10 30 60 120 180 (mins)
pCofilin
CHCHD2
α-tubulin
WT
KO
B
C D C D
pCofilin
CHCHD2
β-actin
- Dox
+ Dox
C
ROCK activity (A.U)
(min) after dissociation
0 50 100 150 200
0 1 2 3
D
WT
KO
0 h
28 h
Area (%)
Time (Hrs)
ns
E
Dox-
Dox+
0hr
28hr
Area (%)
Time (Hrs)
ns
